# Discovery of a Novel Antimicrobial Peptide, Temporin-PKE, from the Skin Secretion of *Pelophylax kl. esculentus*, and Evaluation of Its Structure-Activity Relationships

**DOI:** 10.3390/biom12060759

**Published:** 2022-05-29

**Authors:** Yaxian Lin, Yangyang Jiang, Ziwei Zhao, Yueyang Lu, Xinping Xi, Chengbang Ma, Xiaoling Chen, Mei Zhou, Tianbao Chen, Chris Shaw, Lei Wang

**Affiliations:** 1 School of Pharmacy, Queen’s University Belfast, 97 Lisburn Road, Belfast BT9 7BL, UK; ylin36@qub.ac.uk (Y.L.); yangyang.jiang@qub.ac.uk (Y.J.); zzhao14@qub.ac.uk (Z.Z.); ylu21@qub.ac.uk (Y.L.); x.xi@qub.ac.uk (X.X.); c.ma@qub.ac.uk (C.M.); m.zhou@qub.ac.uk (M.Z.); t.chen@qub.ac.uk (T.C.); chris.shaw@qub.ac.uk (C.S.); 2 School of Traditional Chinese Medicine, Beijing University of Chinese Medicine, Beijing 100029, China

**Keywords:** antimicrobial peptides, temporin, cationic amino acids, D-amino acids, haemolytic activity

## Abstract

Bacterial resistance against antibiotics has led to increasing numbers of treatment failures, and AMPs are widely accepted as becoming potential alternatives due to their advantages. Temporin-PKE is a novel peptide extracted from the skin secretion of *Pelophylax kl. esculentus* and it displays a strong activity against Gram-positive bacteria, with an extreme cytotoxicity. Incorporating positively charged residues and introducing D-amino acids were the two main strategies adopted for the modifications. The transformation of the chirality of Ile could reduce haemolytic activity, and an analogue with appropriate D-isoforms could maintain antimicrobial activity and stability. The substitution of hydrophobic residues could bring about more potent and broad-spectrum antimicrobial activities. The analogues with Lys were less harmful to the normal cells and their stabilities remained at similarly high levels compared to temporin-PKE. The optimal number of charges was three, and the replacement on the polar face was a better choice. Temporin-PKE-3K exerted dually efficient functions includingstrong antimicrobial and anticancer activity. This analogue showed a reduced possibility for inducing resistance in MRSA and *Klebsiella pneumoniae*, a rather strong antimicrobial activity in vivo, and it exhibited the highest therapeutic index such that temporin-PKE-3K has the potential to be developed as a clinical drug.

## 1. Introduction

Infectious diseases have become severe problems for modern public health and have led to a heavier economic burden across the world [1]. In the majority of cases, frequently occurring infections are due to different pathogens, such as urinary tract infections like uncomplicated cystitis and pyelonephritis resulting from *Escherichia coli* (*E. coli*) [2], osteomyelitis and septic arthritis associated with *Staphylococcus aureus* (*S. aureus*) [3], cryptococcosis caused by *Cryptococcus neoformans* [4], and so on. In the past, antibiotics have been utilised to deal with this health problem but increasing multi-drug resistance (MDR) in microbes is occurring due to the overuse and misuse of traditional antibiotics [5]. ESKAPE organisms consist of several bacteria that are highly resistant to antibiotics and cause more treatment failures and higher mortality rates [6]. To combat these pathogens, it is of extreme urgency to find robust and effective substitutes for traditional antibiotics. Antimicrobial peptides (AMPs) have potent and broad-spectrum antimicrobial activity against Gram-negative bacteria, Gram-positive bacteria, and fungi, and they play important roles in the innate immune system [7]. Differing from the mechanisms of traditional antibiotics, which act on specific targets in the microorganisms, AMPs inhibit the growth of pathogens and kill them through multiple mechanisms, including non-specific membrane permeability via various models and acting on intracellular processes so that AMPs are less likely to induce resistance in pathogens compared to antibiotics [8]. Moreover, there are other bioactivities of AMPs, such as anti-inflammatory, anticancer, and insulin-releasing abilities, and these advantages provide AMPs with an extraordinary potential to act as alternatives to antibiotics [9].

AMPs are divided into different families, and temporins constitute one of the most prominent peptide families [10]. The peptides of the temporin family share some common characteristics: they are among the smallest AMPs extracted from amphibians, and their sequence lengths are short, usually 10 to 14 amino acids, with few exceeding to 16–17 residues [11]. The majority contain C-terminal amidation, resulting from post-translational modification, and these hydrophobic peptides tend to form amphipathic α-helical conformations [12]. There are several cationic amino acids in their sequences, such that they present with a net positive charge, usually up to +3, in environments at a neutral pH [13]. In most cases, temporins are active against Gram-positive bacteria while having little or no effect on Gram-negative bacteria. However, a few have been reported to possess activity against Gram-negative bacteria, like temporin-L [12], temporin-1DRa [14], and temporin-SHa [15]. In addition, due to their high hydrophobicity, most temporins display potent cytotoxicity towards normal cells, which is one of the main limitations to the use of AMPs which needs to be solved [16].

In the past, the toxicity of AMPs toward normal cells, which makes them harmful to the host, was one of the main obstacles that prevented their use as clinical drugs [17]. AMPs are usually hydrophobic and this provides the peptides with the ability to permeate cell membranes, which are constituted by phospholipid bilayers [18]. The hydrophobic residues can bind with the inner core of the cellular plasma membrane, and the higher hydrophobicity causes the insertion of the peptides into the membrane [19] such that the peptides have the potential to disrupt the mammalian cell membrane, which leads to their toxicity towards normal cells [20]. Based on previous studies, there are several strategies that can be used reduce cytotoxicity against normal cells. The membranes of bacteria are different from those of mammals and there are more negatively charged components in the bacterial membrane, such as LPS in the outer membrane of Gram-negative bacteria [21]. Therefore, if the peptides are more cationic, they will preferentially interact with the bacterial membrane, which make them less likely to bind with the membrane of normal cells, thus, replacing the hydrophobic amino acids at specific locations with positively charged residues is a practical method for decreasing the cytotoxicity of the peptides [22]. AMPs usually exert antimicrobial activity by forming α-helical conformations when attached to the bacterial membrane [23]. The secondary structure is crucial for the cell selectivity of AMPs, and the higher percentage of α-helix can bring about a stronger haemolytic activity so that by slightly disrupting the α-helical conformation, it is possible to decrease their cytotoxicity against normal cells [24]. The substitution of D-amino acids at particular positions is one of the effective strategies to damage the established secondary structure, and some reports have found that a single replacement with D-isoform residues can reduce the haemolysis of AMPs while maintaining their bioactivity [25]. For instance, a derivative of mellitin, in which several amino acids (V^5^, V^8^, L^11^, and K^16^) were substituted with D-isoforms, displayed a weakened haemolytic activity but an unaffected antimicrobial activity [26]. Without lowering hypotensive activity, the single site substitution of D-lysine in mastoparan B had a significant effect in undermining the cytotoxicity of the peptide [27].

In summary, a novel peptide of the temporin family, isolated from *Pelophylax kl. esculentus*, was named temporin-PKE, and functional and toxicity screenings of a synthetic replicate of this peptide were conducted. In an attempt to reduce the cytotoxicity of the peptide and explore the structure–activity relationships of its potent antimicrobial activity, several modifications were designed and bioactivity assays (antimicrobial assays, antibiofilm assays, and anti-proliferation assays) and cytotoxicity assays, including haemolytic assays and MTT assays against normal cells, were later performed. Multiple different assays were performed with all of the peptides for deeper analyses, including time-killing assays against *S. aureus*, membrane permeability assays, and stability assays in different environments, and the analogue, temporin-PKE-3K, was found to have particular value for further study due to its extraordinary bioactivity and decreased cytotoxicity. Finally, the potential of temporin-PKE and temporin-PKE-3K to induce bacterial resistance was examined, and the antimicrobial activity in vivo was verified in a wax moth larvae model.

## 2. Materials and Methods

### 2.1. The Acquisition of Skin Secretion from Pelophylax kl. esculentus

Specimens of *Pelophylax kl. esculentus* (*n* = 30) were purchased from a commercial source (Le QuerruyCellier, Notre Dame de Monts, France), and were captive bred. They were settled into their new conditions for three months before secretion harvesting. They were kept in our purpose-designed amphibian facility at a temperature of 15–20 °C under a 12 h/12 h light/dark cycle, and they were fed multivitamin-loaded crickets three times per week. Mild transdermal electrical stimulation (5 V), consisting of 3 ms pulses for 30 s duration each, was used to obtain the skin secretion from dorsal skin. This was then washed from the skin with deionised water (dd H_2_O) and snap-frozen in liquid nitrogen. The lyophilizate was stored at −20 °C before further analysis. The procedure for obtaining skin secretion was administered by the Institutional Animal Care and Use Committee (IACUC) of Queen’s University Belfast and approved on 1 March 2011. It was performed in accordance with UK Animal (Scientific Procedures) ACT 1986, Project license PPL 2694, issued by the Department of Health, Social Service and Public Safety, Northern Ireland.

### 2.2. ‘Shotgun’ Cloning of the Precursor-Encoding cDNA of the Novel Peptide

Five milligrams of the lyophilizate from the skin secretion of *Pelophylax kl. esculentus* was used for the isolation of poly-A mRNA using a magnetic Dynabeads^®^ mRNA DIRECT™ Kit (BIOTECH, Bromborough, UK), and the cDNA library was constructed by using a BD SMART™ Rapid Amplification of cDNA Ends (RACE) cDNA Amplification Kit (B.D. Bioscience Clontech, UK). The degenerate primer, which was 5′-GAWYYAYYHRAGCCYAAADATGTTCA-3′ (W = A or T; Y = C or T; H = A or C or T; R = A or G; D = A or G or T), was used in 3′-RACE reactions to clone the products and subsequently the sequencing reaction was conducted.

### 2.3. Isolation and Identification of the Novel Peptide

The skin secretion peptides of *Pelophylax kl. esculentus* were separated by an RP-HPLC system (Waters, Miford, MA, USA), which was fitted with a column (Jupiter C-18, 5 µM, 4.6 mm × 250 mm, Phenomenex, Macclesfield, Cheshire, UK) for 240 min. The molecular masses of collected peptides were analysed by MALDI-TOF MS. The fractions containing molecular masses coincident with the peptide identified from the results of molecular cloning were injected into an LCQ-Fleet electrospray ion-trap mass spectrometer (Thermo Fisher Scientific, San Francisco, CA, USA) to confirm the primary structure of the predicted mature peptide using an MS/MS fragmentation sequencing technique.

### 2.4. Peptide Synthesis

Temporin-PKE and its analogues were synthesised by SPPS, and the whole process was described in detail in a previous paper [28]. The peptides were synthesised by a Tribute^®^ 2-channel peptide synthesiser. The amino groups on the Rink Amide MBHA resin and amino acid were activated by 20% piperidine, and the carboxyl group on the amino acid was activated via HBTU and 11% *N*-methylmorpholine. The resin was coupled with amino acids from C-terminus to N-terminus. Then, a mixture of cleavage solution (94% trifluoroacetic acid (TFA), 2% water, 2% 1,2-ethanedithiol, and 2% thioanisole) of 25 mL/g was added to the crude peptide and stirred at room temperature for at least 2 h. After concentrating, the solution was stored in 50 mL of diethyl ether at −20 °C overnight to precipitate the peptide. After storage, diethyl ether was added into the tube to wash the peptides three times. After air drying, 30 mL of 50/49.95/0.05 (*v*/*v*/*v*) acetonitrile/dd H_2_O/TFA was added to dissolve the sediment, and the peptide solution was placed into a freeze dryer for 48–50 h for lyophilisation.

### 2.5. Purification and Identification of the Synthesised Peptides

The lyophilised peptides were purified by a reverse-phase high-performance liquid-chromatograph (RP-HPLC) system (Cecil Adept 4200, Amersham Biosciences, Buckinghamshire, UK), which was equipped with a Jupiter C-18 column (Phenomenex, Macclesfield, UK) with a size 250 × 21.2 mm. The peptides were eluted by a gradient elution from 100% solution A (99.95% dd H_2_O and 0.05% TFA) to 100% solution B (80% acetonitrile, 19.95% dd H_2_O, and 0.05% TFA) at 6 mL/mL. The wavelength of the HPLC detector was 214 nm. 

The molecular masses of the separated peptides were identified by MALDI-TOF mass spectrometry. The peptides were analysed on a linear time-of-flight Voyager DE mass spectrometer (Perspective Biosystem, Foster City, CA, USA) in positive detection mode using CHCA as the matrix. Two µL of each HPLC elution was loaded and spotted onto the MALDI ground-steel target plate and then air-dried. Afterwards, 1 µL of CHCA matrix solution (10 mg/mL) was loaded to the plate and then air-dried. The peptides were identified by appropriate mass measurements, which depended on their m/z ratios. After purification and identification, the eluted fractions with m/z matching the theoretical molecular mass of the peptides of interest were pumped into the RP-HPLC system fitted with a Jupiter C-5 column (25 × 1 cm, Phenomenex, UK) to examine the purity of each peptide.

### 2.6. Secondary Structure Analyses

The physicochemical properties of temporin-PKE and its analogues were calculated, and their helical wheel projects were performed using HeliQuest (https://heliquest.ipmc.cnrs.fr/, accessed on 1 August 2021). The theoretical molecular masses were calculated by Peptide Mass Calculator from Peptide Protein Research Ltd. (https://www.peptidesynthetics.co.uk/tools/, accessed on 1 August 2021). The precise secondary structures were determined by CD spectroscopy using a JASCO J-815 CD spectrometer (Jasco, Essex, UK). The peptide samples were dissolved in different solutions, including 20 mM NH_4_Ac and 50% TFE in 20 mM NH_4_Ac, at a concentration of 100 µM. The temperature was 20 °C, and the scanning speed was 100 nm/min with 1 nm bandwidth and 0.5 nm data pitch. The wavelengths used for the CD analyses were from 190 nm to 250 nm. The helicity percentages of the peptides were predicted by the online tool, Bestsel (https://bestsel.elte.hu/index.php, accessed on 30 August 2021).

### 2.7. Determination of Minimum Inhibitory (MIC) and Bactericidal (MBC) Concentrations

The antimicrobial activities of peptides were evaluated against the Gram-positive bacteria, *S. aureus* (ATCC 6538), methicillin-resistant *Staphylococcus aureus* (MRSA) (NCTC 12493), *Enterococcus faecalis* (*E. faecalis*) (NCTC 12697); the Gram-negative bacteria, *E. coli* (ATCC CRM 8739), *Klebsiella pneumoniae* (*K. pneumoniae*) (ATCC 43816), *Pseudomonas aeruginosa* (*P. aeruginosa*) (ATCC CRM 9027); and the yeast, *Candida albicans* (*C. albicans*) (ATCC CRM 10231). Clinical isolates of MRSA (B042 V2E1 A), *E. coli* (ATCC BAA-2340), *K. pneumoniae* (ATCC BAA-1705), and *P. aeruginosa* (B004 V2S2 B) were also used to determine their antimicrobial activities. The suspension of microorganisms in the logarithmic growth phase with a specific final density (Gram-negative and Gram-positive bacteria: 5 × 10^5^ CFU/mL; yeast: 1 × 10^6^ CFU/mL) was incubated with different peptide concentrations from 1 µM to 512 µM at 37 °C for 24 h. PBS was used as the negative control, and norfloxacin and amphotericin B were selected as the positive control for bacteria and yeast, respectively. Since the Norfloxacin had weak activities against *P. aeruginosa* (B004 V2S2 B) and *K. pneumoniae* (ATCC BAA 1705), gentamicin was used as the positive control for these two bacterial strains. The absorbance of each well was obtained using a microplate reader (EL808, Biolise BioTek, Winooski, VT, USA) set at 550 nm wavelength. The MIC value was the lowest concentration of a peptide that inhibits visible bacterial growth. Once the MIC values were determined, an aliquot from the bacterial culture exposed to the peptide at or above its MIC value was inoculated on a Mueller Hinton agar (MHA) and was incubated at 37 for 18 h. The MBC value was the lowest concentration of a peptide that kills 99.9% of bacteria.

### 2.8. Haemolysis Assays

As a previous study [29] described in detail, the haemolysis activity assays of peptides were conducted on defibrinated horse blood (TCS Biosciences Ltd., Buckingham, UK) with an erythrocyte suspension concentration of 2% (*v*/*v*). The 120 μL of peptide solution in a gradient concentration ranging from 1µM to 512 µM was mixed with the same volume of 2% horse red blood cell suspension, and the complex was incubated at 37 °C for 2 h. PBS and 0.1% Triton X-100 were chosen as the negative and positive control, respectively. After centrifugation at 900× *g* for 5 min, the absorbance of the supernatant was analysed using a microplate reader (EL808, Biolise BioTek, Winooski, VT, USA) set to a wavelength of 570 nm. The equation to calculate the haemolysis of the peptides is shown below:

$$Haemolysis\;\left(\%\right)=\frac{A-A_{n}}{A_{p}-A_{n}}\times 100\%$$

where *A* represents the absorbance of each treatment group, *A_n_* represents the average absorbance of the negative control group, and *A_p_* represents the average absorbance of the positive control group.

### 2.9. MTT Assays on HaCaT Cells

The cytotoxicity of the peptides against the human epidermal keratinocyte line, HaCaT, was tested using the MTT cell viability assay as described in a previous study [30]. The 100 µL of the cell suspension in a complete DMEM medium with a density of 10,000 cells/mL was seeded in a 96-well plate and incubated overnight at 37 °C in a humidified 5% CO_2_ atmosphere. After 4 h of starvation in a serum-free medium, 100 μL of serial dilutions (from 10^−9^ M to 10^−4^ M) of temporin-PKE and its analogues was added to each well, and the plate was incubated for 24 h. The serum-free medium and 0.1% Triton X-100 were used as the negative and positive control, respectively. Then, MTT solution, at a volume of 10 μL, was added to each well, and the plate was incubated for 2 h. After this, the medium was replaced by 100 μL of DMSO, and plates were put in the shaking incubator for 15 min. The absorbance of each well was measured using a microplate reader (EL808, Biolise BioTek, Winooski, VT, USA) set to a wavelength of 570 nm. The equation used to calculate the viability of the cells is shown below:

$$Cell\;viability\;\left(\%\right)=\frac{A-A_{b}}{A_{n}-A_{b}}\times 100\%$$

where *A* represents the absorbance of each treatment group, *A_b_* represents the average absorbance of the wells in the blank control group (cells not exposed to the test peptide and MTT), and *A_n_* represents the average absorbance of the wells in the negative control group (cells not exposed to the test peptide).

### 2.10. Determination of Minimum Biofilm Inhibitory (MBIC) and Eradication (MBEC) Concentrations

The anti-biofilm activities of the peptides against *S. aureus* (ATCC 6538), MRSA (NCTC 12493), *E. faecalis* (NCTC 12697), *E. coli* (ATCC CRM 8739), *K. pneumoniae* (ATCC 43816), and *P. aeruginosa* (ATCC CRM 9027), were evaluated. For the MBIC assay, 99 µL of bacterial suspension (5 × 10^5^ CFU/mL) was mixed with 1 µL of serial dilutions (from 100 µM to 51,200 µM) of temporin-PKE and its analogues in a 96-well plate, and the plate was incubated in a moist shaking incubator at a speed of 200 rpm at 37 °C for 24 h. For the MBEC assay, 100 µL of bacterial suspension (5 × 10^5^ CFU/mL) in each well was seeded in a new 96-well plate, and the plate was put into the same shaking incubator for 24 h incubation to allow biofilm formation on the well. After biofilm formation, the biofilm in the plate was washed with PBS twice. The PBS was aspirated between washes to remove planktonic bacterial cells. The washed biofilm in each well was treated with 100 µL of serial dilutions (from 1 µM to 512 µM) of the test peptides. Then, the plate was incubated in the same shaking incubator for another 24 h. After being washed by PBS twice and fixed with methanol, the biofilm in each well was incubated with 100 µL of 0.1% crystal violet for 30 min, and the stained biofilm was dissolved in 100 µL of glacial acidic acid after drying. The solutions were transferred to a new microplate, and the absorbance of each well was measured using a microplate reader (EL808, Biolise BioTek, Winooski, VT, USA) set to 595 nm wavelength. The MBIC was the minimum concentration of the peptide showing no biofilm formation. The MBEC is the lowest concentration of the peptide capable of eradicating the mature biofilms, which had no biofilm in the well.

### 2.11. Time-Killing Assays

The antimicrobial efficiency of temporin-PKE and its analogues against *S. aureus* (ATCC 6538) was conducted as described in a previous paper [31]. *S. aureus* in the logarithmic growth phase (OD = 0.23 at 550 nm) was diluted by MHB medium to obtain a final density of 5 × 10^5^ CFU/mL. The peptides with the concentrations of 1×, 2×, and 4× corresponding MIC values were incubated with the bacterial culture for 3 h, and an aliquot was collected from the incubated bacterial culture at 0, 5, 10, 15, 30, 45, 60, 75, 90, 120, 150, and 180 min. The bacteria treated with only MHB medium and 20 μg/mL norfloxacin were used as negative control and positive control, respectively. These collected bacterial cultures were immediately diluted in PBS and inoculated on the MHA plates, and the colonies were counted after overnight incubation at 37 °C. 

### 2.12. Membrane Permeability Assays

The SYTOX Green Nucleic Acid Stain (Life Technologies, Carlsbad, CA, USA) was applied for the evaluation of the potency of temporin-PKE and its analogues to permeate the inner membrane of *S. aureus* and *E. coli* [32]. *S. aureus* (ATCC 6538) and *E. coli* (ATCC CRM 8739) in the logarithmic growth phase were subcultured in TSB and LB, respectively, at 37 °C for 2.5 h. After incubation, the bacterial culture was centrifuged at 1000× *g* for 10 min at 4 °C to collect the cell pellet. After washing with 5% TSB or LB in 0.85% NaCl solution twice, the bacterial cell pellet was resuspended in 5% TSB or LB to reach a certain OD value (OD*_S. aureus_* = 0.68–0.72, OD*_E. coli_* = 0.65–0.70 at 590 nm) which corresponds to 1 × 10^8^ CFU/mL. A volume of 50 μL of the bacterial suspension was mixed with 40 μL of the peptide solution at concentrations of 1×, 2×, and 4× MIC against *S. aureus* and *E. coli* in a black 96-well plate, and the plate was incubated at 37 °C for 2 h. In addition, equivalent quantities of cells treated with 5% TSB or LB in 0.85% NaCl solution were used as the negative control. Melittin (8 μM in a final concentration) and 70% isopropanol were utilised as a positive control for *S. aureus* and *E. coli,* respectively. After incubation, 10 μL of 10% SYTOX Green dye was added to each well, and the plate was put into the shaking incubator for 5–10 min, and the fluorescent intensity was analysed by an ELISA plate reader (EL808, Biolise BioTek, Winooski, VT, USA) with excitation at 485 nm and emission at 528 nm.

### 2.13. Salt and Serum Sensitivity Assays

According to the procedures in a previous article [33], the analysis of the sensitivity of the peptides was determined by measuring MIC values in the presence of different types of salts and 10% fetal bovine serum. MRSA (NCTC 12493) and *K. pneumoniae* (ATCC 43816) were selected as the subjects of these assays. Various salts at different concentrations, including 150 mM NaCl, 4.5 mM KCl, 6 μM NH_4_Cl, 1 mM MgCl_2_, 1 mM MgCl_2_, 8 mM ZnCl_2_, 2.5 mM CaCl_2_, 4 mM FeCl_3_, and 10% serum (Gibco, Auckland, New Zealand), were added to the MHB medium for the examination of the MIC values which were determined as described in Section 2.7.

### 2.14. Anti-Proliferation Assays

The anti-proliferative activities of peptides against HCT-116, NCI-H838 (H838), U251MG, PC-3, and NCI-H157 cell lines were determined by MTT cell viability assay [31], and the details were described in Section 2.9.

### 2.15. Resistance Induction Assays

To test the possibility of temporin-PKE inducing resistance in MRSA and temporin-PKE-3K inducing resistance in both MRSA and *K. pneumoniae*, a method published by another research group [34], was followed. Firstly, the MIC of each peptide against MRSA and *K. pneumoniae* was determined and recorded as described in Section 2.7. Then, 100 µL of a bacterial suspension at a concentration of 1/2 MIC was aspirated and inoculated into a bottle of fresh MHB, and the MIC assay was conducted once again using this inoculum. After 24 h incubation, 200 µL of bacterial suspension from the previous passage was harvested for the following inoculation and then incubated for another 24 h. This process was repeated for 15 cycles, and the MIC values of the peptides against bacteria were tested every three passages and recorded.

### 2.16. Determination of Antimicrobial Activity In Vivo

The antimicrobial activities of temporin-PKE and temporin-PKE-3K, in vivo, were studied in the wax moth larvae model [30]. Wax moth larvae (Livefood UK Ltd., Rooks Bridge, UK), weighing 250 ± 25 mg, were divided into different groups (*n* = 9 in each group). Ten µL of MRSA suspension, at a density of 5 × 10^6^ CFU/mL, was injected into each larva. After 2 h of exposure, 10 µL of peptide solution, with concentrations of 4, 8, and 16 mg/kg, was injected into the larvae in each group. PBS was utilised as the negative control, and 50 mg/kg vancomycin was selected as the positive control for MRSA. The survival rates of the larvae were recorded every 12 h for 120 h. 

### 2.17. Statistical Analyses

All data from the assays on bioactivity evaluation were analysed using Prism (Version 6.0; GraphPad Software Inc., San Diego, CA, USA). The results from the assays in vitro were analysed by two-way ANOVA and Dunnett’s multiple comparisons test. The results from the assays in vivo were analysed by survival analysis and curve comparison tests. The significances were indicated by **** (*p* < 0.0001) and *** (*p* < 0.001).

## 3. Results

### 3.1. Molecular Cloning of the Novel Precursor-Encoding cDNA

**Figure 1:**
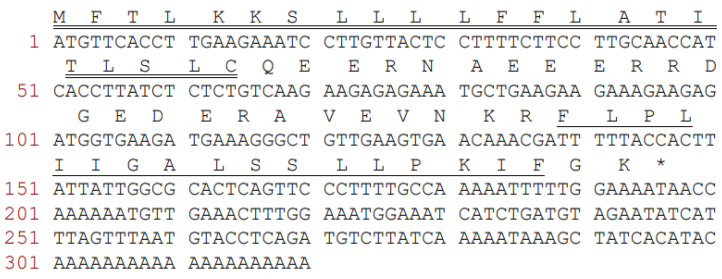
The nucleotide and the translated open-reading frame amino acid sequence of the precursor-encoding cDNA of a novel peptide, temporin-PKE, was cloned from *Pelophylax kl. esculentus* skin secretion. The putative signal and mature peptides are underlined by a bold line and a single line, respectively. An asterisk indicates the stop codon. The full nucleotide sequence is numbering on the left-hand side.

A full-length cDNA, which encoded the biosynthetic precursor of temporin-PKE, was successfully cloned from the cDNA library of the skin secretion from the frog, *Pelophylax kl. esculentus* (Figure 1). The open reading frame of the novel peptide consisted of 65 amino acid residues, divided into four domains. The putative signal peptide domain contained 22 residues as indicated by the NCBI-BLAST analysis. Then, this was followed by a hydrophilic spacer peptide of 22 residues, rich in acidic amino acids, and this ended in a highly-conserved twin basic residue site, Lys-Arg (-KR-). The mature peptide was composed of 17 residues followed by a –GK dipeptide sequence, which indicated the presence of an amidation at the C-terminal of this peptide. Therefore, the deduced sequence of the novel peptide was: FLPLIIGALSSLLPKIF-NH_2_. The nucleotide sequence of peptide temporin-PKE was deposited in the GenBank database under the accession number ON382518.

### 3.2. Novel Peptide Identification and Sequence Analysis

**Figure 2:**
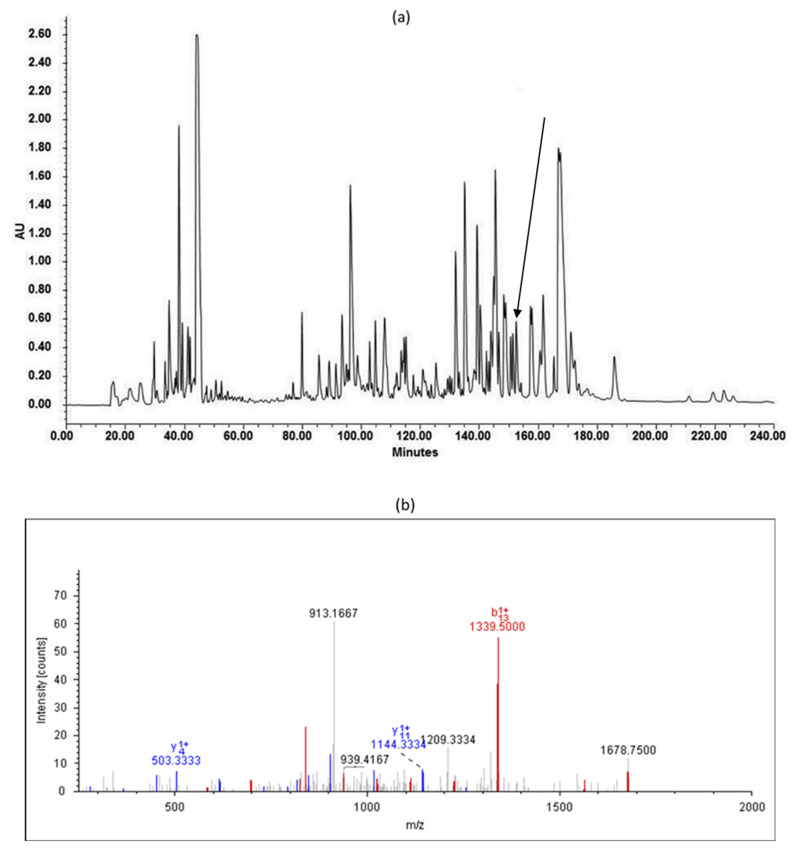

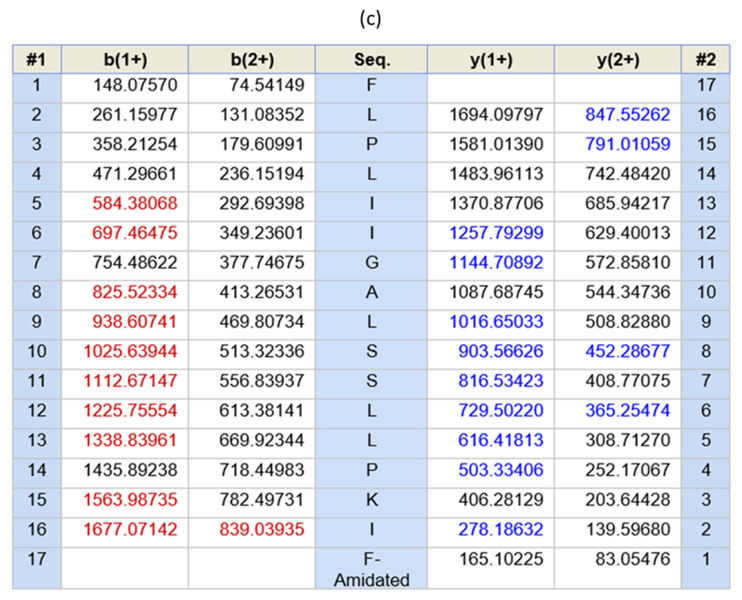
(**a**) The RP-HPLC chromatogram of the skin secretion of *Pelophylax kl. esculentus*. The peak of temporin-PKE is indicated by an arrow. (**b**) The annotated LCQ tandem mass (MS/MS) fragmentation spectrum of temporin-PKE. Prominent ions in are labeled with the observed m/z value and ion series designation. b and y denote the N- and C-terminal fragments of the peptide produced by breakage at the peptide bond in LCQ, respectively. The number represents the residue number from either the N- or C- terminus. (**c**) Electrospray ion-trap MS/MS fragmentation data derived from fragment ions in panel (**b**) corresponding in molecular mass to temporin-PKE. Predicted singly- and doubly-charged ions are coloured black. The actual b-ions and y-ions detected by MS/MS fragmentation are indicated in red and blue typeface, respectively.

**Figure 3:**
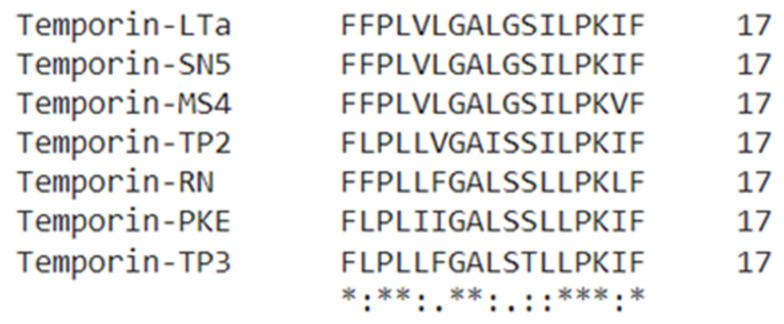
The multiple sequence alignments between the novel peptide and several peptides in the temporin family with the same sequence length by Clustal Omega. An * (asterisk) indicates that the residue in this position in these sequences is fully conserved. A : (colon) indicates conservation between amino acids with strongly similar properties. A . (period) marks conservation between residues of weakly similar properties. The numbers at the ends of the sequences are the numbers of the amino acids.

The skin secretion of *Pelophylax kl. esculentus* was fractionated by RP-HPLC (Figure 2a). The molecular mass (Figure 2b) and the primary structure (Figure 2c) of the peptide in the selected fraction were determined by LCQ tandem mass spectrometric (MS/MS) fragmentation. The molecular mass of the novel peptide was found to be 1841 Dalton (Da), and the sequence was consistent with that deduced from the molecular cloning. Compared to some peptides in the temporin family (Figure 3), there were many similarities in the sequence of amino acids. The novel temporin peptide contained a C-terminal amidation, which is a common feature of peptides in the temporin family to which this peptide belongs. As Table 1 shows, peptides in the temporin family contained diverse bioactivities, including broad-spectrum antimicrobial activity, antibiofilm activity, and anticancer activity, so the novel peptide, temporin-PKE, which belongs to temporin family, is likely to possess these bioactivities. Therefore, temporin-PKE was selected as the main subject of this research for the following bioactivity studies.

### 3.3. Peptide Design

**Figure 4:**
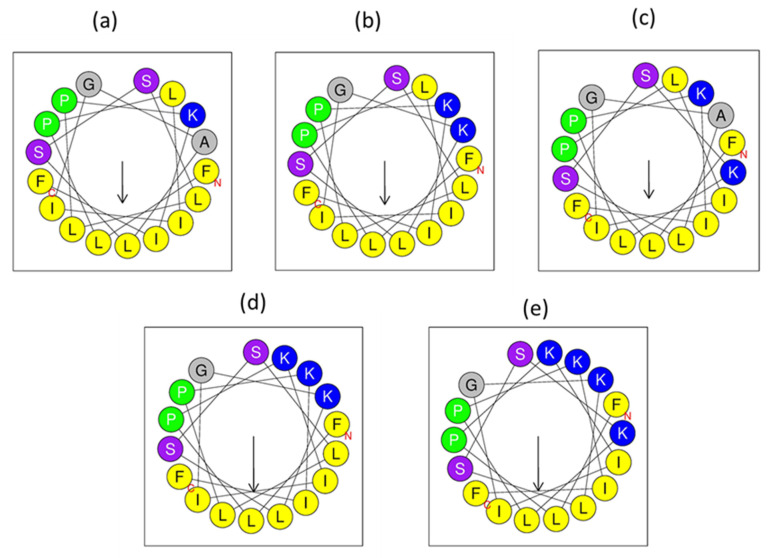
The helical wheel representations of (**a**) temporin-PKE, (**b**) temporin-PKE-2K, (**c**) temporin-PKE-K_12_, (**d**) temporin-PKE-3K, and (**e**) temporin-PKE-4K, as predicted by HeliQuest. The hydrophobic, hydrophilic, and positively charged amino acids are coloured in yellow, purple, and blue, respectively.

The hydrophobicity index of temporin-PKE is 1.068, which is relatively high, and this can lead to strong haemolytic activity against normal blood cells. The introduction of positively charged residues and the replacement of non-natural amino acids, like D-isomers, are the two main strategies to decrease the haemolysis activity and retain the bioactivity at the same time [43,44]. Therefore, different numbers of hydrophobic amino acids in the various positions of the temporin-PKE sequence were substituted with lysine. To explore the influence of different sites in the sequence, the alanine^8^ on the polar face and the leucine^12^ on the hydrophobic face (Figure 4) were replaced by lysine and the two analogues were named temporin-PKE-2K and temporin-PKE-K_12_, respectively (Table 2). Their respective hydrophobicity values were reduced to 0.992 and 0.910, and the HM of temporin-PKE-2K reached 0.513, which is the highest among these three peptides. More residues of lysine were added into the sequence: based on the sequence of temporin-PKE-2K, the leucine^4^ on the hydrophilic face was replaced to design a new derivative, temporin-PKE-3K, and then, the leucine^12^ was substituted by lysine, and this peptide was named temporin-PKE-4K. With the increase in the numbers of lysine residues, the hydrophobicity of the analogues decreased dramatically, and due to the disruption of the hydrophobic face, the HM of temporin-PKE-4K was slightly lower than that of temporin-PKE-3K. To lower the hydrophobicity, the hydrophobic amino acid, isoleucine, was chosen to be substituted by its D-form. The Ile^16^ was replaced by D-Ile, and the resulting peptide was named, temporin-PKE-i, and in another peptide, all the Ile resides were modified into D-amino acids, and this peptide was named temporin-PKE-3i. 

### 3.4. Purification and Identification of the Analogues

Temporin-PKE and its analogues were synthesised by SPPS, and they were purified by HPLC (Figure S1). The molecular masses of the peptides were identified by MALDI-TOF MS (Figure S2). The molecular masses obtained were entirely consistent with those predicted, such that it could be concluded that the synthesised peptides were authentic temporin-PKE and its designed derivatives.

### 3.5. Secondary Structure Analyses

**Figure 5:**
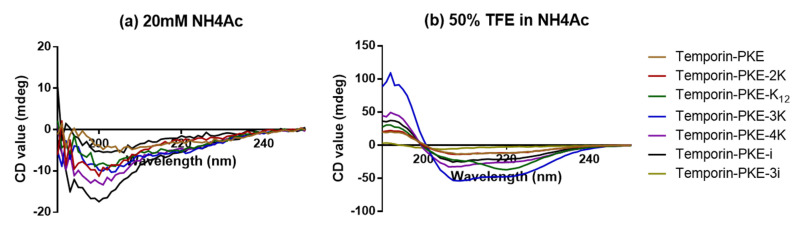
The CD spectra of temporin-PKE and its analogues in (**a**) aqueous solution (20 mM NH_4_Ac) and (**b**) membrane-mimicking solution [50% (*v*/*v*) trifluoroethanol (TFE) in NH_4_Ac].

The precise secondary structures of temporin-PKE and its analogues were determined by CD, and in the aqueous environment (such as 20 mM NH_4_Ac), temporin-PKE and its analogues were presented as random coils (Figure 5). The addition of cationic amino acids improved the proportion of α-helix, except for temporin-PKE-K_12_ (Table 3). The temporin-PKE-2K can form a 20.3% α-helical structure in 50% TFE in NH_4_Ac, which is similar to that of the parent peptide, while the percentage of the α-helical structure of temporin-PKE-3K in the membrane-mimicking environment was the highest among all the analogues, which reached 72.8%. Damage to the hydrophobic face had a great influence on the secondary structures, such that temporin-PKE-4K only adopted 44.6% α-helix when it was exposed to 50% TFE in NH_4_Ac, which is much lower than that of temporin-PKE-3K. The introduction of different quantities of D-residues has a diverse influence on the ability to form α-helical conformations. The temporin-PKE-i retained its capacity to adopt α-helix in 50% TFE, while temporin-PKE-3i could not form an α-helical structure since the proportion of α-helix in aqueous and membrane-mimicking solution was 0.0 and 0.8%, respectively.

### 3.6. Antimicrobial Assays

The antimicrobial activity of temporin-PKE is targeted specifically at the membrane of Gram-positive bacteria, which was similar to that of the positive control, and can combat selected bacteria at a relatively low concentration, such as at 4 µM against clinically isolated MRSA (Table 4). The analogues with D-amino acids still had no activity to inhibit the growth of Gram-negative bacteria even at the highest test concentration, while different numbers of D-amino acids had a distinctive influence on activity. The effect of temporin-PKE-i was similar to the parent peptide and the MICs of temporin-PKE-3i against Gram-positive bacteria, including clinical isolates, were much higher than temporin-PKE. However, differing from the original peptide, temporin-PKE-3i had a modest ability against yeast and could inhibit the growth of *C. albicans* at 128 µM. Overall, the substitution of hydrophobic residues with Lys resulted in broad-spectrum antimicrobial activity, and when the total number of positive charges was over three, the peptide achieved the ability to attack yeast. The antimicrobial activity of temporin-PKE-2K against Gram-positive bacteria was modestly promoted compared to the parent peptide, and the MIC against *E. faecalis* was even lower than that of norfloxacin. Moreover, temporin-PKE-2K attained the capacity to kill gram-negative bacteria and can combat the chosen clinical isolates at a relatively low concentration. However, the MICs of temporin-PKE-2K against gram-negative bacteria were much higher than those of the selected antibiotics. With the same total number of cationic charges, the capacity against Gram-positive bacteria of temporin-PKE-K_12_ was slightly weaker than that of temporin-PKE-2K, and the MIC values against Gram-negative bacteria were mainly twofold higher than temporin-PKE-2K. With the increasing number of Lys, temporin-PKE-3K maintained potent antimicrobial ability against the majority of the tested gram-positive bacteria at 2 µM, which demonstrated a comparable antimicrobial activity to norfloxacin. In addition, the antimicrobial activity of temporin-PKE-3K against the Gram-negative bacteria *K. pneumoniae* and *P. aeruginosa* showed a dramatic improvement, but the MICs were slightly higher than the MICs of the positive control. Additionally, temporin-PKE-3K attained a modest inhibitory effect against *C. albicans* at 32 µM, which was 8-fold higher than the MIC of amphotericin B. Unfortunately, with the addition of an extra Lys, the antimicrobial activity of temporin-PKE-4K was undermined to a large extent, and when compared to temporin-PKE-K_12_, although temporin-PKE-4K’s capacity against Gram-positive bacteria was much weaker, it was slightly enhanced against Gram-negative bacteria. Moreover, the MIC of temporin-PKE-4K showed a 2-fold increase over that of temporin-PKE-3K.

The HC_50_ of temporin-PKE was 6.58 µM, which revealed a strong haemolysis activity against red blood cells, such that its TI against Gram-positive bacteria was relatively low, at just 1.96 (Table 4). The TI values of the analogues were increased to different degrees, which illustrated the greater safety of these peptides. In terms of the Gram-positive bacteria, the modifications of the amino acids in the hydrophobic face led to higher TI values, except for temporin-PKE-3i, and the highest TI was temporin-PKE-4K which achieved 62.10. The highest overall TI still belongs to temporin-PKE-4K, and the TI of temporin-PKE-3K was 20.53, which is considered a relatively high value.

### 3.7. Toxicity

#### 3.7.1. Haemolysis Activity

**Figure 6:**
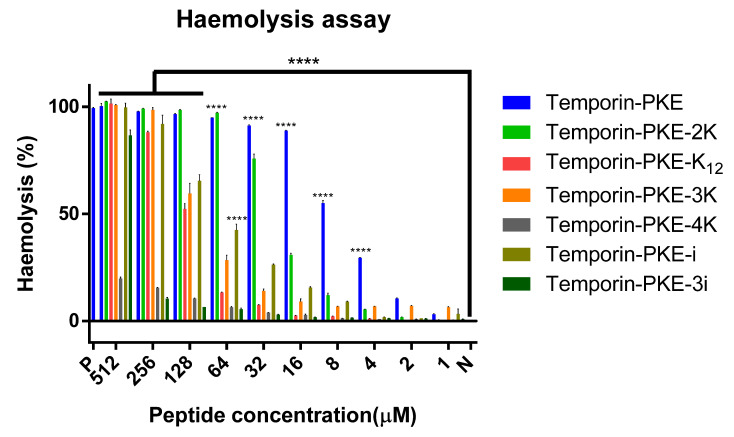
The haemolytic activities of temporin-PKE and its analogues. N represents the negative control and P represents the positive control, PBS and 0.1% Triton X-100 in this experiment, respectively. The results were analysed by two-way ANOVA and Dunnett’s multiple comparisons test that compared haemolysis of various concentrations of peptides with the negative control group. The significance is indicated by ****, which represents *p* < 0.0001. The error bar represents the SEM of the nine replicates from three independent tests.

Temporin-PKE displayed a potent haemolysis activity, with an HC_50_ of just 6.58 µM (Table 4). With the introduction of positively charged amino acids, the toxicity of the analogues against red blood cells was reduced to different degrees (Figure 6). The HC_50_ of temporin-PKE-2K was 3-fold higher than that of temporin-PKE, and temporin-PKE-3K could destroy 50% of red blood cells at a concentration of 87.47 µM, which is much higher than its working concentrations. The replacement of the leucine in the hydrophobic face gave temporin-PKE-4K and temporin-PKE-K_12_ much weaker haemolytic activity, with HC_50_ values of 1671 and 122.7 µM, respectively. The addition of the D-amino acid made temporin-PKE-i and temporin-PKE-3i less toxic towards red blood cells than the original peptide.

#### 3.7.2. Cytotoxicity on HaCaT Cells

**Figure 7:**
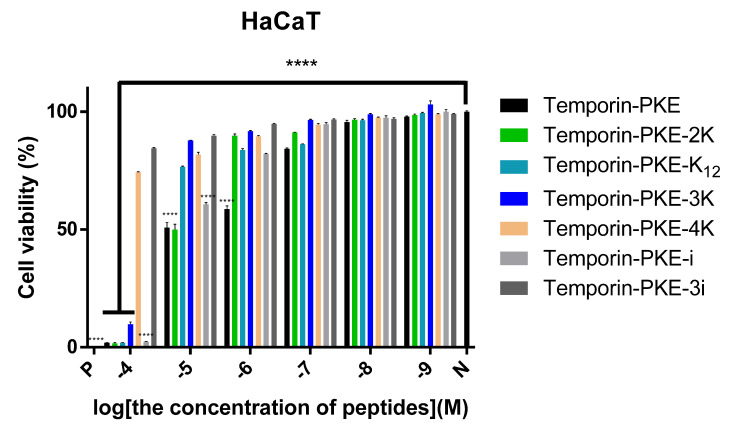
The cytotoxicity of temporin-PKE and its designed derivatives on HaCaT cells. N represents the negative control and P represents the positive control, which were PBS and 0.1% Triton X-100 in this experiment, respectively. The results were analysed by two-way ANOVA and Dunnett’s multiple comparisons test compared the cell viability of different concentrations of peptides with that of the negative control group. The significance is indicated by **** which indicates *p* < 0.0001. The error bar represents the SEM of the nine replicates from three independent tests.

Apart from temporin-PKE-4K and temporin-PKE-3i, other peptides led to low cell viability of these normal cells at the highest test concentration (10^−4^ M), and temporin-PKE exhibited greater cytotoxicity against HaCaT with a rather low IC_50_ (Figure 7). Differing from the results of the haemolysis assay, temporin-PKE-K_12_ and temporin-PKE-i tended to damage 50% of HaCaT cells at low concentrations, with IC_50_ values of 18.99 and 11.9 µM, respectively (Table 5). Temporin-PKE-3K displayed modest cytotoxicity with an IC_50_ against the normal cell line of 29.56 µM, which is much higher than its MICs.

### 3.8. Antibiofilm Activity

Similar to their antimicrobial activities, temporin-PKE, temporin-PKE-i, and temporin-PKE-3i had no effect on the biofilms of the selected Gram-negative bacteria, but they could inhibit the formation of biofilm for the Gram-positive bacteria at a relatively low concentration, except for temporin-PKE-3i (Table 6). Temporin-PKE and temporin-PKE-i could remove the mature biofilm formed by *S. aureus* and MRSA while they did not influence the mature biofilm of *P. aeruginosa*. With a total number of two positive charges, temporin-PKE-2K and temporin-PKE-K_12_ displayed similar anti-biofilm activities and they could eradicate the mature biofilm of *S. aureus* and MRSA at a modest concentration while their MBECs against other bacteria were over 512 µM. With the introduction of an extra Lys, the MBICs and MBECs of temporin-PKE-3K against all the chosen bacteria were much lower than the other analogues, and they obtained the ability to eradicate the mature biofilm of Gram-negative bacteria. However, the antibiofilm activity of temporin-PKE-4K was dramatically weakened compared to temporin-PKE-3K. 

### 3.9. Bacteria Killing Efficiency

**Figure 8:**
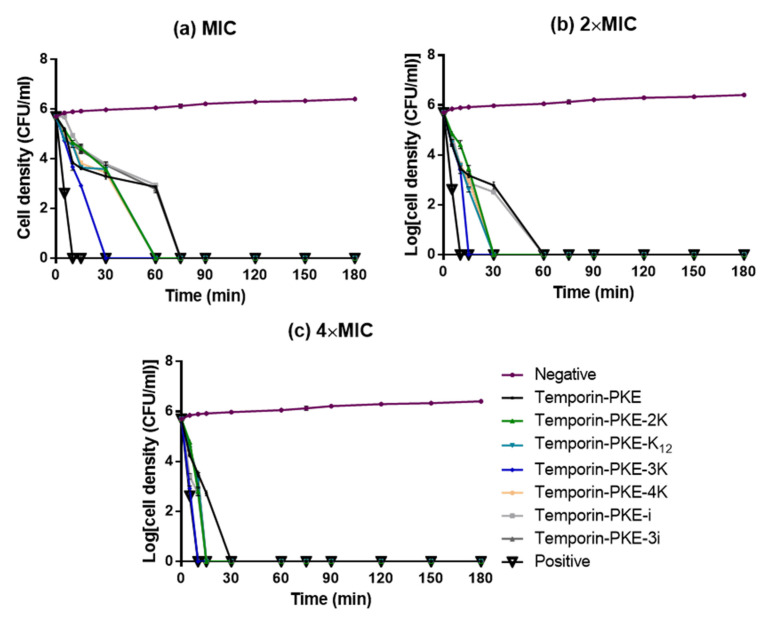
The kinetic time-killing curves of temporin-PKE and its analogues against *S. aureus* at the concentrations of (**a**) 1 × MIC, (**b**) 2 × MIC, and (**c**) 4 × MIC. Bacteria treated with MHB medium only and 20 μg/mL norfloxacin were used as the negative and positive control, respectively. The error bar represents the SEM of the three replicates from three independent tests.

In general, the killing efficiency of temporin-PKE against *S. aureus* showed a tendency of ‘fast-slow-fast’ at different concentrations, and the time spent to kill *S. aureus* became smaller with the increase of the test concentration of peptides (Figure 8). Temporin-PKE-i and temporin-PKE-3i had similar antimicrobial efficiencies to the original peptide, and they required 75 min to kill *S. aureus* at their MICs. The analogues with the additional positive charges required less time to damage the *S. aureus* cells, and this decreased 2-fold when increasing the concentration. The antimicrobial efficiency of temporin-PKE-3K was the most potent among these peptides and it only required 5 min to totally destroy cells of *S. aureus* at a concentration of 8 µM, indicating that the analogue had a similar antimicrobial efficiency to the positive control.

### 3.10. Membrane Permeability

**Figure 9:**
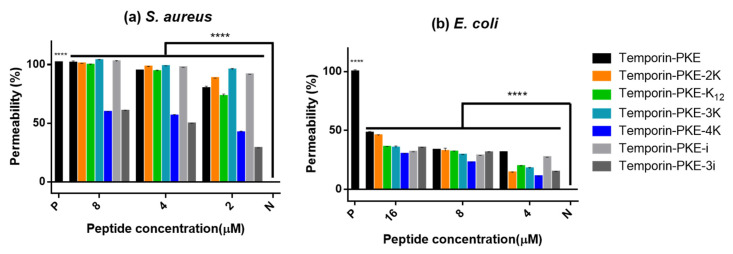
The membrane permeability of temporin-PKE and its analogues against (**a**) *S. aureus* and (**b**) *E. coli*. N represents the negative control, which is 5% TSB in 0.85% NaCl and 5% LB in 0.85% NaCl, respectively. P represents the positive control, which is 8µM mellitin and 70% Isopropanol, respectively. The results were analysed by two-way ANOVA and Dunnett’s multiple comparisons test compared the cell viability of different concentrations of peptides with that of the negative control group. The significance is indicated by **** which means *p* < 0.0001. The error bar represents the SEM of the nine replicates from three independent tests.

In terms of the mechanism of antimicrobial activity against *S. aureus*, the membrane permeabilities of the peptides at various concentrations, which were selected according to the MIC values, had a significant difference from that of the negative control group (Figure 9), which revealed that they killed *S. aureus* via disrupting the membrane. Temporin-PKE-4K and temporin-PKE-3i displayed a lower permeability than other peptides because the test concentrations were much lower than their MICs (32 µM). With *E. coli*, the figures illustrate that they indeed perturbed the membrane to kill the bacteria while the permeability of the inner membrane was much lower than that against *S. aureus* due to the presence of the outer membrane in the Gram-negative bacteria.

### 3.11. Salt and Serum Sensitivities 

The MICs of temporin-PKE and its analogues against MRSA and *K. pneumoniae* were increased to different levels in environments with various salts and serums (Table 7 and Table 8). Temporin-PKE-2K, temporin-PKE-3K, and temporin-PKE-i exhibited excellent stability in the chosen environments with a 2-fold increase for most of their MICs, while they were least stable in the presence of 8 µM ZnCl_2_. Consistent with the antimicrobial activity, most of the MICs of temporin-PKE-3i against MRSA reached 256 µM and 512 µM, which were 4- and 8-fold increases over its original MIC. The analogues with the additional positive charges exhibited similar stability against *K. pneumoniae* as they did against MRSA. In contrast, they were more stable in the environment with 10% FBS and 8 µM ZnCl_2_, with a 4-fold increase and constant MICs, respectively. However, temporin-PKE-K_12_ and temporin-PKE-4K were least stable when in the presence of Ca^2+^ because there was an observed 8-fold increase in their MICs against *K. pneumoniae*. 

### 3.12. Anti-Cancer Cell Proliferattive Activity 

**Figure 10:**
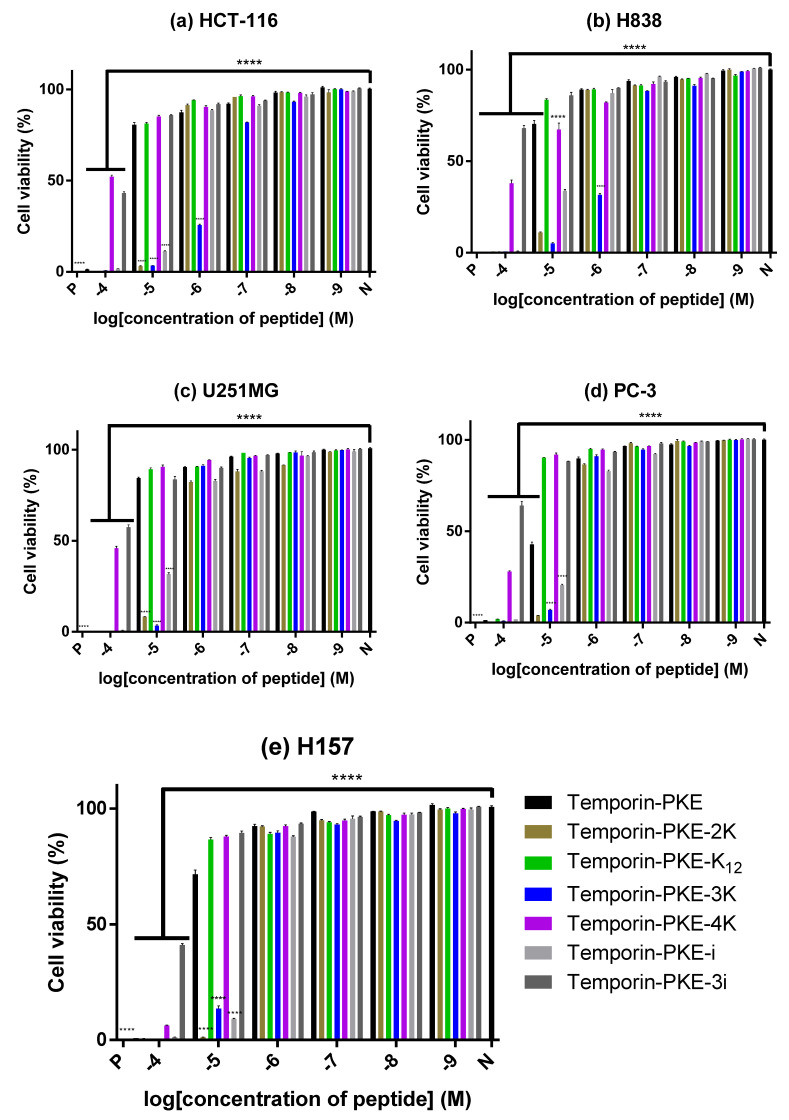
The anti-proliferation activity of temporin-PKE and its analogues against (**a**) HCT-116, (**b**) H838, (**c**) U251MG, (**d**) PC-3, and (**e**) H157 cells. PBS and 0.1% Triton X-100 were used as negative control and positive control, respectively. The results were analysed by two-way ANOVA and Dunnett’s multiple comparisons test, which compared different concentrations of peptides with the negative control group. The symbol **** indicates *p* < 0.0001. The error bar represents the SEM of the nine replicates in three tests (three replicates in each test).

Temporin-PKE-4K and temporin-PKE-3i showed relatively weak anti-proliferative activity against the selected cancer cell lines while they showed comparatively great inhibitory ability against H157 cells, with a relatively low cell viability at the concentration of 10^−4^ M (Figure 10). Temporin-PKE and one of its analogues, temporin-PKE-K_12_, exhibited similar activity to inhibit the proliferation of most cancer cells, except for PC-3 cells, for which the IC_50_ of the parent peptide was just 7.29 µM (Table 9). The anti-proliferative activity of temporin-PKE-2K and temporin-PKE-3K against these chosen cancer cell lines was much more potent than other peptides, with low IC_50_ values. Practically, temporin-PKE-3K could inhibit 50% of the proliferation of H838 cells and HCT-116 cells at concentrations of 0.51 and 0.38 µM, respectively, which revealed the great value of temporin-PKE-3K in developing a potential anti-cancer agent.

### 3.13. Resistance Induction of MRSA and K. pneumoniae

**Figure 11:**
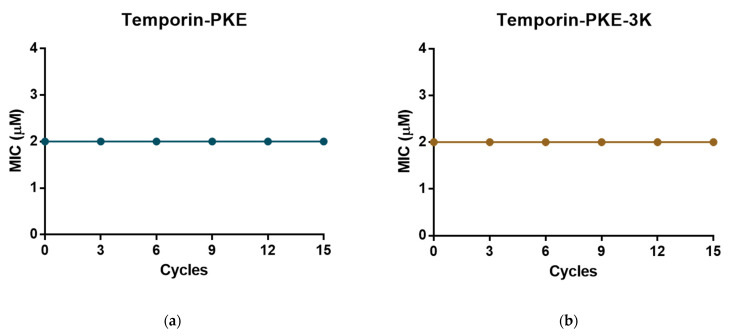
The assessment of resistance induction of (**a**) temporin-PKE and (**b**) temporin-PKE-3K in MRSA after 15 cycles. The vertical axis represents the MIC value (µM) against MRSA, and the horizontal axis means the number of passages.

**Figure 12:**
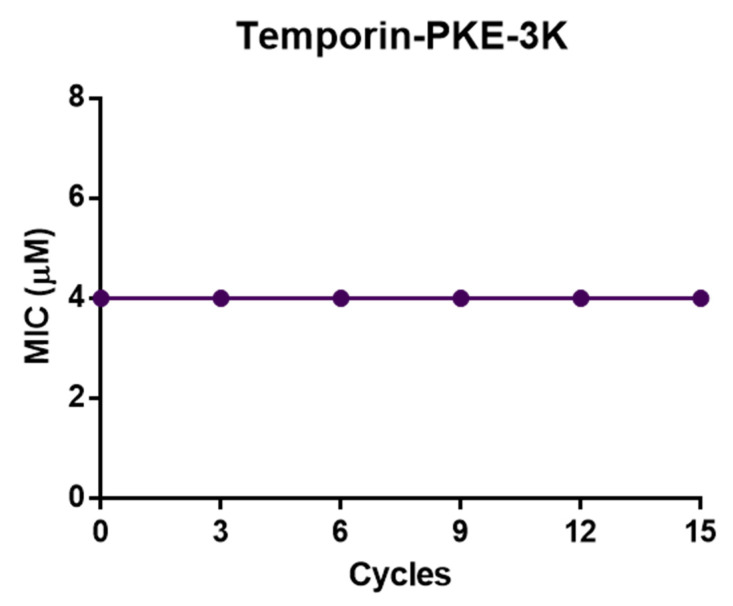
The assessment of the resistance induction of temporin-PKE-3K in *K. pneumoniae* after 15 cycles. The vertical axis represents the MIC value (µM) of temporin-PKE-3K, and the horizontal axis represents the number of passages.

The original peptide and temporin-PKE-3K possessed potent antimicrobial activity against Gram-positive bacteria, and their MICs against MRSA were 2 µM. After six tests of the MICs in the bacterial suspension incubated with the peptide, the MICs of these two peptides stayed constant (Figure 11), which demonstrated that temporin-PKE and temporin-PKE-3K did not induce resistance in MRSA. The MIC of temporin-PKE-3K against *K. pneumoniae*, which is a highly virulent and antibiotic-resistant Gram-negative bacterium, was 4µM, and this remained unchanged against the bacteria after 15 cycles of cultivation with the peptide (Figure 12) so that the temporin-PKE-3K displayed no resistance induction in *K. pneumoniae*.

### 3.14. Determination of Antimicrobial Activity In Vivo

**Figure 13:**
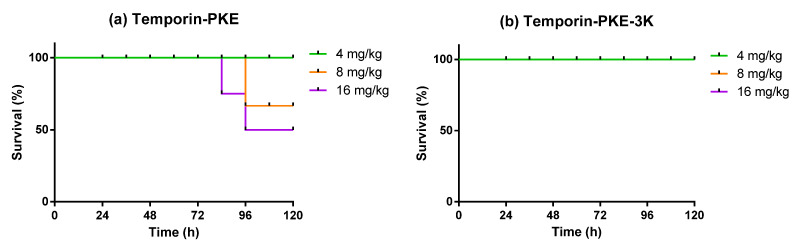
The curves of the survival rate of the waxworm larvae treated with (**a**) temporin-PKE and (**b**) temporin-PKE-3K.

**Figure 14:**
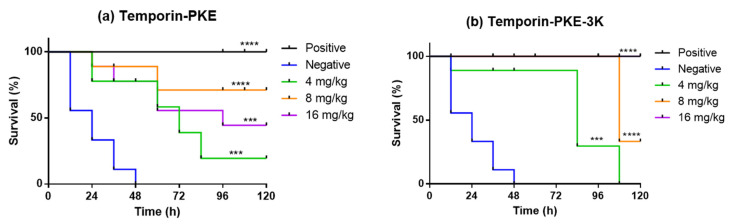
The curves of survival of (**a**) temporin-PKE and (**b**) temporin-PKE-3K in wax worm larvae infected by MRSA. PBS and 50 mg/kg Vancomycin were used as the negative control and positive control, respectively. The data were analysed by the survival analysis and curve comparison test comparing the survival rate of different concentrations of peptides with that of the negative control group. The significance was indicated by **** (*p* < 0.0001) and *** (*p* < 0.001).

The antimicrobial activities of temporin-PKE and temporin-PKE-3K against MRSA in vivo were examined in the wax moth larvae model. The parent peptide exhibited modest toxicity against *Galleria mellonella* larvae in the late stage with 50% mortality at the highest test concentration (Figure 13). In contrast, under doses of 16 mg/kg, the wax moth larvae totally survived injection with temporin-PKE-3K such that this peptide showed no harm to the larvae at the selected concentrations. When the two peptides were utilised to treat larvae infected with MRSA, temporin-PKE at concentrations of 8 mg/kg and 16 mg/kg displayed a similar survival rate in the early stage owing to their comparable efficacy in killing MRSA. However, the final survival rate of the peptide with a concentration of 8 mg/kg, which was up to 89%, was higher than that of the highest test concentration due to the stronger toxicity of temporin-PKE with 16 mg/kg (Figure 14). The low survival rate of the parent peptide with 4 mg/kg resulted from the fact that the concentration was not enough to combat MRSA in vivo. The analogue, temporin-PKE-3K, with the most negligible dose, had no activity to halt infection by MRSA, and it resulted in high mortality of 67% at 8 mg/kg within the last 12 h. Fortunately, the antimicrobial activity in vivo of temporin-PKE-3K at a concentration of 16 mg/kg, was similar to that of 50 mg/kg vancomycin, which was the positive control in this assay, and it caused 100% survival of larvae within the examined 120 h.

## 4. Discussion

Bacteria cause many diseases that threaten the health of people worldwide, and severe bacterial infections lead to a high mortality rate [46]. Antibiotics are substances that are active against pathogens, and they are considered one of the most successful drugs used to heal bacterial infections and have saved millions of lives throughout the world [47]. With the increased usage of antibiotics, which most pathogens were susceptible to initially, high degrees of resistance in the microorganisms ensued which were acquired through mutations or by horizontal gene transfer (HGT) [48]. The majority of the genes involved in the mutations encode for the targets or the transporters of the antibiotics, and the genes passed through HGT did not exist in the human pathogens before the appearance of antibiotics and mainly originated from commensal and environmental bacteria [49]. AMPs have been considered as alternatives to traditional antibiotics to combat pathogens due to their advantages [50]. Living in an environment surrounded by many microorganisms, the AMPs from amphibians are one of the most significant components of the innate immune system to protect the host against these invaders [51]. AMPs possess broad-spectrum antimicrobial activity against different types of bacteria, including Gram-negative and Gram-positive bacteria, fungi, etc. [52]. Owing to the mechanism of direct membrane disruption, the AMPs show a lower possibility of inducing resistance when compared to antibiotics, which kill the bacteria by acting on specific targets [53]. In addition, there are many other bioactivities of AMPs, such as their important roles in immunomodulation [54], anti-inflammatory activity [55], and anticancer activity [56].

*Pelophylax kl. esculentus*, which is known as the edible frog, is a common species from Europe, and it is a hybrid of the pool frog (*Pelophylax lessonae*) and the marsh frog (*Pelophylax ridibundus*) [57]. The AMPs from the skin secretion of *Pelophylax kl. esculentus* have a more significant influence on the immune system to defend against invaders, and they have more potent anti-fungal activity and stability than those peptides from the parent frogs such that they are deserving of further research [58]. The AMPs extracted from the skin secretion of this frog are mainly brevinins, esculentins, and temporins, which possess potent effects against microorganisms [59]. For instance, temporin-PE displays a more potent antimicrobial activity against different kinds of pathogens than other peptides extracted from *Pelophylax kl. esculentus* [11]. Temporin-PKE was one of the peptides extracted from the skin secretion of *Pelophylax kl. esculentus,* and after analysing the primary structure of this peptide, it was determined to belong to the temporin family. Sharing the common characteristics of peptides in the temporin family, temporin-PKE displays a significantly potent antimicrobial activity against Gram-positive bacteria and has no effect on Gram-negative bacteria while its strong haemolysis activity gives it a rather low therapeutic index. Therefore, the main purpose of this project was to decrease the haemolytic activity of the novel peptide and maintain its bioactivity at the same time.

The introduction of cationic residues and D-amino acids were two main strategies adopted to reduce the haemolysis activity of temporin-PKE. The replacements of different amounts of positively charged amino acids at different positions greatly influenced its antimicrobial activity and selectivity. The results of these modifications demonstrated that the analogues with an appropriate number of positive charges were beneficial for the formation of secondary structures and the capacity to combat different kinds of bacteria. It is well known that the initial step of the mechanism of AMPs is the electrostatic interaction between the peptide and the membrane [60], and one of the main distinctions between the membranes of Gram-negative and Gram-positive bacteria is that the former contains a higher proportion of negatively charged components [61]. The parent peptide had only one cationic charge in its sequence, which made it less likely to be attracted to Gram-negative bacteria, so this was one of the causes of its high specificity towards Gram-positive bacteria. With the addition of cationic residues, the spectrum of the analogues temporin-PKE-2K, -K_12_, -3K, and -4K against pathogens became broader, revealing that the extra charges enabled these peptides to strongly interact with the membranes of Gram-negative bacteria. The potential for temporin-PKE-2K and temporin-PKE-3K to form an α-helix was improved compared to the original peptide, and particularly, the percentage of α-helix of temporin-PKE-3K was raised to 72.8%, which is a nearly 4-fold increase over that of temporin-PKE. Meanwhile, substituting hydrophobic residues on the polar face with Lys, as in temporin-PKE-2K and -3K, led to a higher amphipathicity, which gave the peptide more probability to adopt an α-helix conformation. When the peptides are attached to the bacterial membrane, they start to transfer their conformation into the α-helix, and the higher amphipathicity displays a much greater distribution of hydrophobic and hydrophilic residues, which enables the peptide to bind to the membrane [62]. Therefore, the analogues, temporin-PKE-2K and -3K, achieved a more potent activity to permeate the bacterial membrane, which resulted in a stronger and more broad-spectrum antimicrobial activity, especially against Gram-negative bacteria and fungi. However, increasing the number of cationic amino acids blindly cannot enhance the antimicrobial activity of the peptide due to the decreased hydrophobicity, which causes a lowering of the insertion into the bilayer phospholipid of the membrane [63]. Compared to temporin-PKE-3K, temporin-PKE-4K contains a higher number of cationic charges, but it leads to a much lower hydrophobicity. Therefore, it has a much lower activity in inserting into the bacterial membrane so that the antimicrobial activity against bacteria is much weaker than temporin-PKE-3K. Moreover, the position of the charges is also an important feature of their antimicrobial activity. Based on the results, the disruption of the hydrophobic face, which brings about a decreased possibility of forming an α-helix, weakens the ability to inhibit the growth of bacteria or kill them because the lower amphipathicity is not beneficial for the interaction between the peptide and the hydrophobic core of the membrane. In comparison, in temporin-PKE-K_12_ and -4K, the amino acid on the hydrophobic face was replaced by Lys, while their antimicrobial activity against different types of bacteria changed. The high hydrophobicity of temporin-PKE-K_12_ resulted in a stronger ability to combat Gram-positive bacteria compared to the original peptide, while temporin-PKE-4K possessed greater numbers of charges, which enabled it to bind with the membrane of the Gram-negative bacteria. Therefore, finding the balance between the charges and the hydrophobicity of the peptide and replacing the amino acids with an appropriate number of Lys is significant for modifications to AMPs. The optimal number of charges of temporin-PKE is three, and the substitution of the residues on the polar face is a better choice for the rational design to enhance the antimicrobial activity of temporin-PKE. In addition, the introduction of a D-amino acid on the hydrophobic face has a great effect on the secondary structure of the peptides, and greater amounts of D-isoforms cannot lead to a more interesting conformation [64]. The addition of one isoleucine into temporin-PKE had a minor influence on the formation of an α-helix so that the activity against Gram-positive bacteria for temporin-PKE-i was similar to the parent peptide while the replacement of all the Ile largely disrupted the secondary structure of the peptide, which brought about a loss of the majority of the antimicrobial activity. Nevertheless, the activity of temporin-PKE-i and -3i could not extend the antimicrobial spectrum against both Gram-negative and Gram-positive bacteria because of the constant number of cationic amino acids.

The toxicity of AMPs, which results in a low TI value, is one of the main obstacles that hinder their development for clinical use, and it is the high hydrophobicity of AMPs that makes them more readily interactive with mammalian membranes [65]. It is well known that there are many negatively charged components on the bacterial membrane, while the outer layer of the mammalian membrane only contains zwitterionic phospholipids and other neutral elements [66]. With an increase in the number of charges, the selectivity of the analogues is promoted to a large degree because the peptides with more cationic residues are more likely to be attracted to the anionic bacterial membrane rather than the neutral membrane of normal cells. Moreover, temporin-PKE-K_12_ and -4K, whose hydrophobic faces were damaged, exhibited lower haemolysis activity due to their decreased potential to form an α-helix and their lower amphipathicity, making it harder for them to insert themselves into the mammalian membrane. Based on the same principle, the analogues with D-amino acids, temporin-PKE-i and -3i, also disrupted their hydrophobic faces and their lower amphipathicity led to decreased interaction with the membranes. The higher selectivity of these analogues gave them larger TI values, which revealed that they were worthy of further study and development into clinical drugs due to their much higher selectivity and thereby greater safety.

The SYTOX Green staining assay revealed that the mechanism of temporin-PKE and its analogues against *S. aureus* and *E. coli* was permeation of the bacterial membrane. The permeability of the membrane of Gram-negative bacteria was much lower than that of Gram-positive bacteria because the peptide needed to translocate across the outer membrane, which is a unique structure on the membrane of Gram-negative bacteria, as the first step. It is widely accepted that the AMPs that adopt the direct disruption of the bacterial membrane as their antimicrobial mechanism will accumulate at the surface of the membrane and then transfer into its specific conformation, and when the peptides/lipid ratio reaches a certain threshold, the peptides can interact with the bacterial membrane to kill the bacteria [67]. The hydrophobic residues will bind to the hydrophobic core of the membrane, and the polar amino acids tend to connect with the hydrophilic head groups on the bilayer phospholipids [68]. The amphipathicity, which allocates the hydrophilic and hydrophobic amino acids at opposite sides, benefits the interaction between the peptides and cell membranes [69]. Although the percentages of membrane permeability for these peptides were similar, temporin-PKE-3K, which contains the highest amphipathicity, displayed the strongest activity leading to the lysis of the bacterial membrane. Furthermore, the optimal number of charges and the highest amphipathicity, which facilitate the peptide to be attracted to and interact with the membrane, gave temporin-PKE-3K the highest efficiency against *S. aureus* among these peptides consistent with the most potent antimicrobial activity and the most increased membrane permeability.

Biofilm is one of the main causes of chronic infectious diseases, and it can lead to resistance to antibiotics [70]. During the formation of biofilm, pathogens will produce a matrix to facilitate the colonisation of the surface, and this becomes a massive barrier that hinders the entrance of the drugs so that many agents fail to eliminate the biofilm and kill the pathogens [71]. The peptides usually exert their antibiofilm activity via killing the bacteria directly so that the MBICs are similar to the MICs against the same bacteria. The matrix in the biofilm contains many negatively charged components, such as EPS, DNA, and proteins, so the peptides with several positively charged residues can bind to them and bring about the eradication of the biofilm [72]. Therefore, the increase of the cationic amino acids gives the analogues temporin-PKE-2K, -K_12_, -3K, and -4K an enhanced ability to inhibit the growth of the biofilm and eradicate the mature biofilm, especially temporin-PKE-3K, which exhibited low MBECs against many multiple-antibiotic resistant bacteria. 

Cancers, which are diseases caused by the uncontrollable growth and spread of the body’s cells, are hard to cure through modern medical technologies, and cause high mortality, and thus more effective anticancer agents urgently need to be developed [73]. Similar to the constitution of the bacterial membrane, there are abundant anionic biomolecules, like PS, O-glycosylated mucins, heparan sulphate, and so on accumulated on the outer layers of the cancer cell membranes [74]. This study revealed that temporin-PKE-3K, with increased charges compared to the original peptide, has a greater antiproliferative activity against HCT-116 cells with an IC50 value of 0.38 µM and lower cytotoxicity against normal human keratinocytes with an IC50 value of 29 µM. Thus, this analogue has some potential to be an anticancer agent. Furthermore, it has been revealed that bacteria can be one of the causes of cancers and some cancers are usually accompanied by bacterial infections [75]. For example, *Helicobacter pylori* is a bacterium that was discovered to induce gastric cancer and extra-gastric cancer in humans [76]. Many previous researchers reported that the occurrence of infection caused by MRSA in most cancer patients is relatively high [77]. Therefore, a peptide with the dual function of antimicrobial activity and anticancer activity, such as temporin-PKE-3K, has a high potential value for more in-depth research.

MRSA and *K. pneumoniae* are two main bacteria in the group of ESKAPE pathogens, which represent several leading causes of infections due to their multidrug resistance which causes a high percentage of treatment failures [78]. Temporin-PKE and its analogue, temporin-PKE-3K, could maintain constant MIC values when incubated with these two bacteria, which demonstrated that they contain a decreased possibility of inducing resistance in MRSA and *K. pneumoniae*. Moreover, the two peptides display relatively high stability against MRSA and *K. pneumoniae* in environments with different salts and serum and their antimicrobial activity against MRSA was verified in vivo, especially for temporin-PKE-3K, which could provide a 100% survival rate in infected wax moth larvae at a relatively low concentration. It is widely accepted that when the TI value is more than 10, the drug will not be considered a narrow therapeutic index drug [79]. In comparison, the TI value of temporin-PKE-3K was 20.53, which is much higher than the parent peptide. Given all the advantages of temporin-PKE-3K, including potent bioactivities, high efficiency, high selectivity, strong stability, and a decreased possibility of inducing resistance, this peptide possesses an elevated potential for becoming a clinical drug to treat the diseases caused by MRSA and perhaps other ESKAPE pathogens.

## 5. Conclusions

Through the analogue design of temporin-PKE, a novel peptide was discovered in the skin secretion of *Pelophylax kl. esculentus*; several structure-activity relationships have been elucidated, as with the increase of the positive charges within a certain amount; and temporin-PKE’s antimicrobial activity and selectivity were enhanced dramatically. The position of cationic amino acids is also a critical factor along with the disruption of the hydrophobic face affecting the secondary structure which thus leads to weaker bioactivity. The introduction of D-amino acid substitutions into this peptide can indeed decrease the undesirable toxicity while causing no improvement in the desired bioactivity. Among all the analogues designed, peptide temporin-PKE-3K could be considered an excellent analogue for further clinical studies due to its many functional advantages.

## Figures and Tables

**Table 1:** The sequences and bioactivities of several peptides in the temporin family.

Peptide Name	Species	Sequence	Bioactivities
temporin-LTa	*Hylarana latouchii*	FFPLVLGALGSILPKIF-NH_2_	Antimicrobial activity against gram-positive bacteria [35]
temporin-1CEa	*Rana chensinensis*	FVDLKKIANIINSIFGK-NH_2_	Broad-spectrum antimicrobial activity and antitumour activity [36]; anti-inflammatory activity [37]
temporin-MS1	*Hylarana maosuoensis*	FLTGLIGGLMKAL-NH_2_	Broad-spectrum antimicrobial activity [38]
temporin G	*Rana temporaria*	FFPVIGRILNGIL-NH_2_	Antiviral activity against influenza and parainfluenza respiratory viruses [39]
temporin L	*Rana temporaria*	FVQWFSKFLGRIL-NH_2_	Potent and broad-spectrum antimicrobial activity, especially against gram-negative bacteria [40]; Antibiofilm activity [41]; Antiendotoxic activity [42]

**Table 2:** The physicochemical properties of temporin-PKE and its analogues.

Peptide	Sequence	Hydrophobicity <H>	^1^ HM <μH>	Net Charge (z)	^2^ Theoretical MM (g/mol)	^2^ Observed MM (g/mol)
Temporin-PKE	FLPLIIGALSSLLPKIF-NH_2_	1.068	0.490	+1	1841	1841
Temporin-PKE-2K	FLPLIIGKLSSLLPKIF-NH_2_	0.992	0.513	+2	1898	1898
Temporin-PKE-K_12_	FLPLIIGALSSKLPKIF-NH_2_	0.910	0.443	+2	1856	1856
Temporin-PKE-3K	FLPKIIGKLSSLLPKIF-NH_2_	0.834	0.657	+3	1913	1913
Temporin-PKE-4K	FLPKIIGKLSSKLPKIF-NH_2_	0.675	0.644	+4	1928	1928
^3^ Temporin-PKE-i	^4^ FLPLIIGALSSLLPKiF-NH_2_	-	-	+1	1841	1841
^3^ Temporin-PKE-3i	^4^ FLPLiiGALSSLLPKiF-NH_2_	-	-	+1	1841	1841

^1^ HM is the abbreviation of hydrophobic moment, which is a measure of the amphipathicity of a helix [45]. ^2^ MM is the abbreviation of the molecular mass. ^3^ The hydrophobicity and HM of temporin-PKE-i and temporin-PKE-3i cannot be analysed by Heliquest due to the presence of D-amino acids. ^4^ The lower-case letters represent the D-amino acids of Isoleucine.

**Table 3:** Secondary structure analysis of temporin-PKE and its analogues.

Peptide	20 mM NH_4_Ac			50% TFE in NH_4_Ac		
Name	Helix (%)	Antiparallel (%)	Turn (%)	Helix (%)	Antiparallel (%)	Turn (%)
Temporin-PKE	4.4	35.3	15.4	18.3	27.9	13.1
Temporin-PKE-2K	0.9	36.0	15.6	20.3	18.2	13.4
Temporin-PKE-K_12_	3.1	39.2	14.0	6.8	8.7	7.1
Temporin-PKE-3K	5.0	24.7	15.3	72.8	0.0	2.8
Temporin-PKE-4K	2.8	38.7	14.2	44.6	3.2	11.7
Temporin-PKE-i	5.5	34.8	14.8	35.5	8.5	13.4
Temporin-PKE-3i	0.0	46.0	14.8	0.8	40.9	14.6

**Table 4:** The MIC and MBC values of temporin-PKE and its analogues against several pathogens ^1^.

Bacteria	MIC ^2^/MBC ^3^ (μM)							
	Positive ^4^	Temporin-PKE	2K	K_12_	3K	4K	i	3i
*S. aureus* (ATCC 6538)	2/2	2/2	2/2	4/4	2/2	32/32	2/2	32/32
*E. coli* (ATCC CRM 8739)	1/1	>512	4/4	8/8	2/2	4/4	>512	>512
*C. albicans* (ATCC CRM 10231)	4/4	>512	>512	>512	32/64	64/64	>512	128/256
MRSA (NCTC 12493)	2/2	2/2	2/2	4/4	2/2	32/32	4/4	64/64
*K. pneumoniae* (ATCC 43816)	2/2	>512	16/32	32/64	4/4	16/16	>512	>512
*P. aeruginosa* (ATCC CRM 9027)	2/2	>512	128/256	256/256	16/16	64/64	>512	>512
*E. faecalis* (NCTC 12697)	4/4	8/8	2/2	8/8	2/2	32/32	4/4	128/128
Clinical strains								
MRSA (B042 V2E1 A)	2/2	4/8	2/2	4/4	2/2	16/16	4/4	64/64
*E. coli* (ATCC BAA-2340)	2/2	>512	8/8	16/16	2/2	32/64	>512	>512
*P. aeruginosa* (B004 V2S2 B)	1/1	>512	32/32	64/64	8/8	32/32	>512	>512
*K. pneumoniae* (ATCC BAA-1705)	2/2	>512	16/16	64/64	8/8	32/32	>512	>512
HC_50_ (μM)		6.58	19.87	122.7	87.47	1671	68.37	64.0
G^+^ bacteria GM (μM)		3.36	2.00	4.76	2.00	26.91	3.36	32.00
Overall GM ^5^ (μM)		-	7.46	17.15	4.26	26.49	-	-
TI (Gram^+^ bacteria)		1.96	9.94	25.79	43.74	62.10	20.33	6.20
TI ^5,6^ (Overall)		-	2.66	7.16	20.53	63.08	-	-

^1^ The results were achieved from the 15 replicates in the three independent assays. ^2^ The MIC was the lowest concentration at which no bacterial growth could be observed. ^3^ The MBC was the lowest concentration without any bacterial colonies, which meant at this concentration, the peptide reduced the viability of the pathogen by more than 99.9%. ^4^ The positive control for *C. albicans* (ATCC CRM 10231) was amphotericin B. Gentamicin acted as the positive control for *P. aeruginosa* (B004 V2S2 B) and *K. pneumoniae* (ATCC BAA 1705). Norfloxacin was used as the positive control for other bacterial strains in the table. ^5^ As the antimicrobial activities of temporin-PKE, temporin-PKE-i, and temporin-PKE-3i were specifically against Gram-positive bacteria, their overall GM and TI scores were not calculated. ^6^ TI is the ratio between HC_50_ (µM) and the GM of MICs (µM), which represents the antimicrobial selectivity of the peptides, which is descried in a previous study [25].

**Table 5:** The IC_50_ values of temporin-PKE and its analogues against HaCaT cells.

Normal Cell Lines	IC_50_ (µM)						
	Temporin-PKE	2K	K_12_	3K	4K	i	3i
HaCaT	5.04	9.04	18.99	29.56	250.6	11.90	497.1

**Table 6:** The MBIC and MBEC values of temporin-PKE and its analogues.

Bacteria	MBIC ^1^/MBEC ^2^ (μM)						
	Temporin-PKE	2K	K_12_	3K	4K	i	3i
*S. aureus* (ATCC 6538)	2/256	2/64	4/128	2/64	32/128	2/128	64/128
*E. coli* (ATCC CRM 8739)	>512	64/>512	64/>512	16/512	128/>512	>512	>512
*K. pneumoniae* (ATCC 43816)	>512	128/>512	512/>512	32/512	256/>512	>512	>512
*P. aeruginosa* (ATCC CRM 9027)	>512	>512	>512	64/512	128/>512	>512	>512
MRSA (NCTC 12493)	4/256	2/64	4/256	2/128	32/512	2/128	64/>512
*E. faecalis* (NCTC 12697)	32/>512	16/>512	64/>512	4/>512	256/>512	4/>512	128/>512

^1^ The MBIC was the minimum concentration of the peptide showing no biofilm formation. ^2^ The MBEC is the lowest concentration of the peptide that can eradicate the mature biofilms, which had no biofilm in the well.

**Table 7:** The MICs of temporin-PKE and its analogues against MRSA in the presence of various salts and 10% FBS.

Peptide	MIC (μM)								
	150 mM NaCl	4.5 mM KCl	6 µM NH_4_Cl	8 µM ZnCl_2_	1 mM MgCl_2_	2 mM CaCl_2_	4 µM FeCl_3_	10% FBS	Negative
Temporin-PKE	8	8	8	32	8	8	8	16	2
2K	8	4	4	16	4	4	2	8	2
K_12_	32	32	8	32	16	16	8	32	4
3K	8	4	4	16	4	4	2	4	2
4K	128	32	32	128	32	64	32	128	32
i	4	2	2	16	2	4	2	8	2
3i	512	512	256	512	256	256	128	512	64

**Table 8:** The MICs of temporin-PKE and its analogues against *K. pneumoniae* in the presence of various salts and 10% FBS.

Peptide	MIC (μM)								
	150 mM NaCl	4.5 mM KCl	6 µM NH_4_Cl	8 µM ZnCl_2_	1 mM MgCl_2_	2 mM CaCl_2_	4 µM FeCl_3_	10% FBS	Negative
Temporin-PKE	>512	>512	>512	>512	>512	>512	>512	>512	>512
2K	128	16	16	16	32	32	16	64	16
K_12_	128	32	32	64	128	256	32	128	32
3K	16	4	4	4	16	16	16	16	4
4K	64	16	16	16	64	128	16	64	16
i	>512	>512	>512	>512	>512	>512	>512	>512	>512
3i	>512	>512	>512	>512	>512	>512	>512	>512	>512

**Table 9:** The IC_50_ values of temporin-PKE and its derivatives against several cancer cell lines.

Cancer Cell Lines	IC_50_ (µM)						
	Temporin-PKE	2K	K_12_	3K	4K	i	3i
U251MG	23.24	2.49	25.75	2.83	85.52	4.46	120.6
PC-3	7.29	2.64	27.32	3.01	49.50	3.35	167.0
H838	16.38	3.07	22.67	0.51	35.28	5.28	193.4
HCT-116	21.31	2.84	22.09	0.38	99.36	3.07	72.06
H157	17.40	2.78	24.03	3.29	27.76	2.94	71.18

## Data Availability

The nucleotide sequence of peptide temporin-PKE was deposited in the GenBank database under Accession Number ON382518.

## Supplementary Materials

The following supporting information can be downloaded at: https://www.mdpi.com/article/10.3390/biom12060759/s1, Figure S1: The RP-HPLC chromatograms of temporin-PKE and its analogues; Figure S2: The mass spectra of temporin-PKE and its analogues obtained from MALDI-TOF MS.
