# Supplementary material for: Discovery of a Novel Antimicrobial Peptide, Temporin-PKE, from the Skin Secretion of Pelophylax kl. esculentus, and Evaluation of Its Structure-Activity Relationships
Source: Biomolecules. 2022 May 29;12(6):759. doi: 10.3390/biom12060759 (PMC9221509; doi:10.3390/biom12060759)
Supplement: Supplementary file 1 [file biomolecules-12-00759-s001.zip › biomolecules-1732773-supplementary.pdf]

Supplementary Material

Figure S1. The RP-HPLC chromatograms of (a) temporin-PKE, (b) temporin-PKE-2K, (c) temporin-PKE-K_{12}, (d) temporin-PKE-3K, (e) temporin-PKE-4K, (f) temporin-PKE-i and (g) temporin-PKE-3i. The elution peak of each peptide is indicated by an arrow.

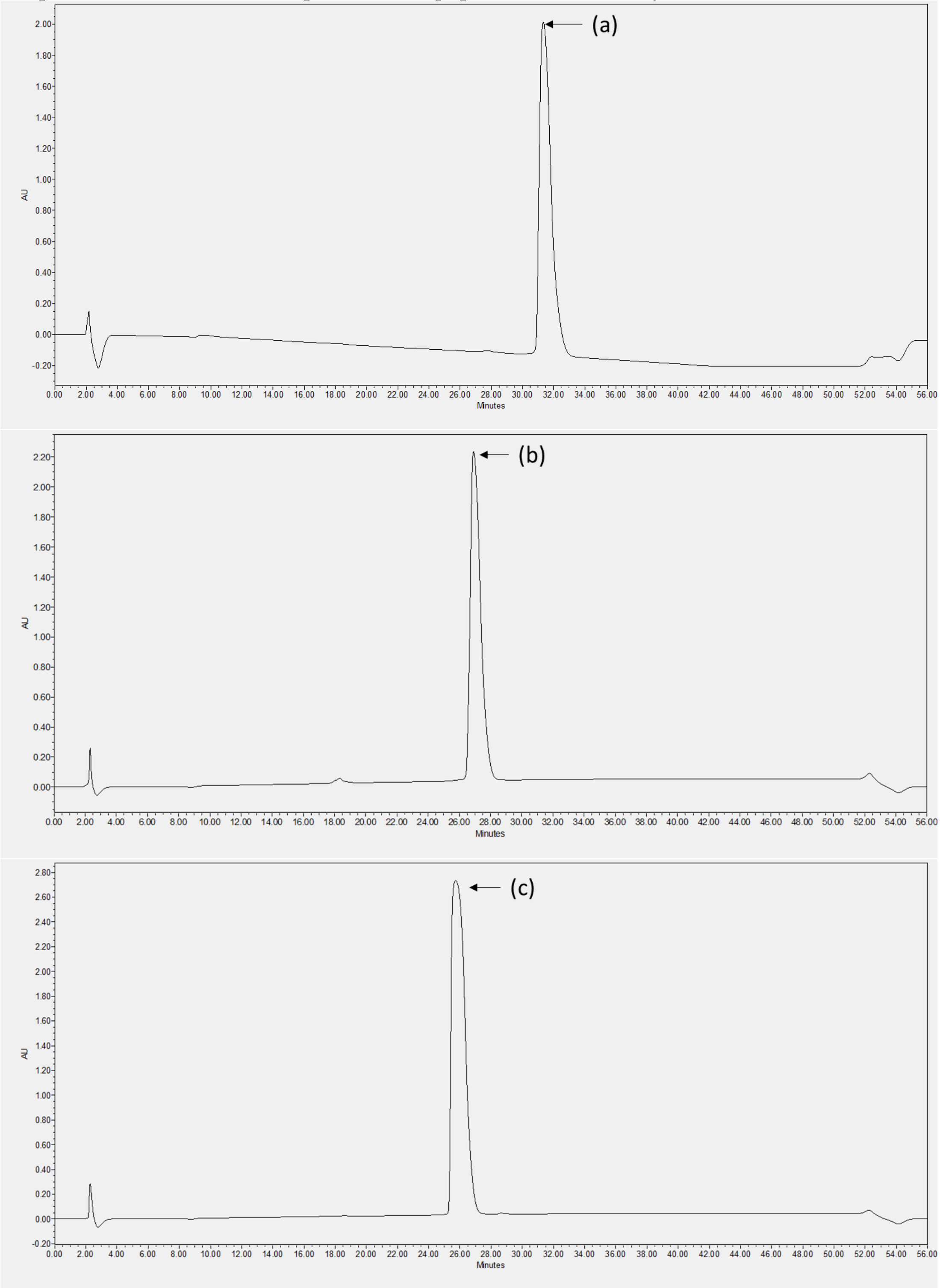

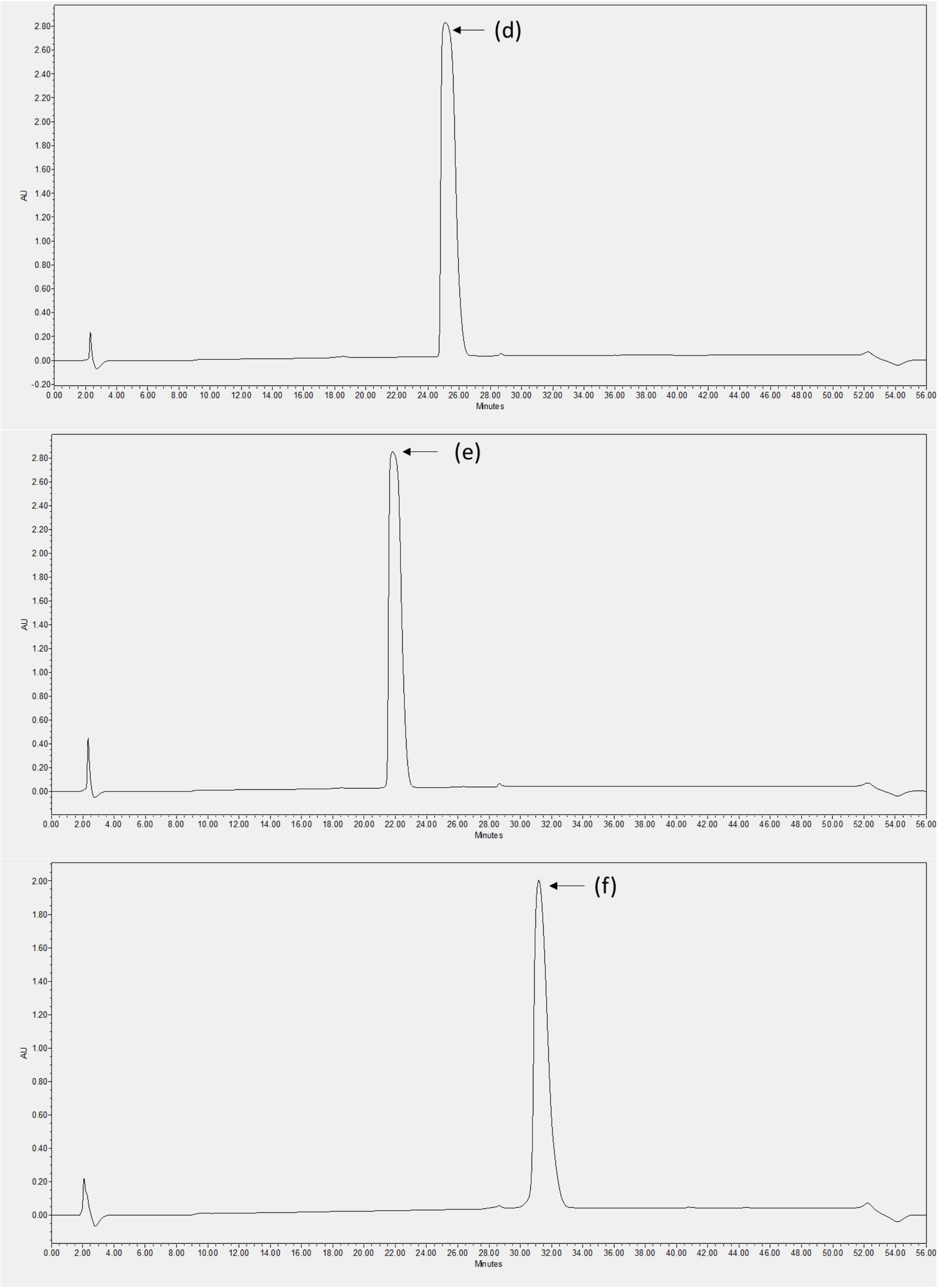
(d)
AU
Minutes
(e)
AU
Minutes
(f)
AU
Minutes

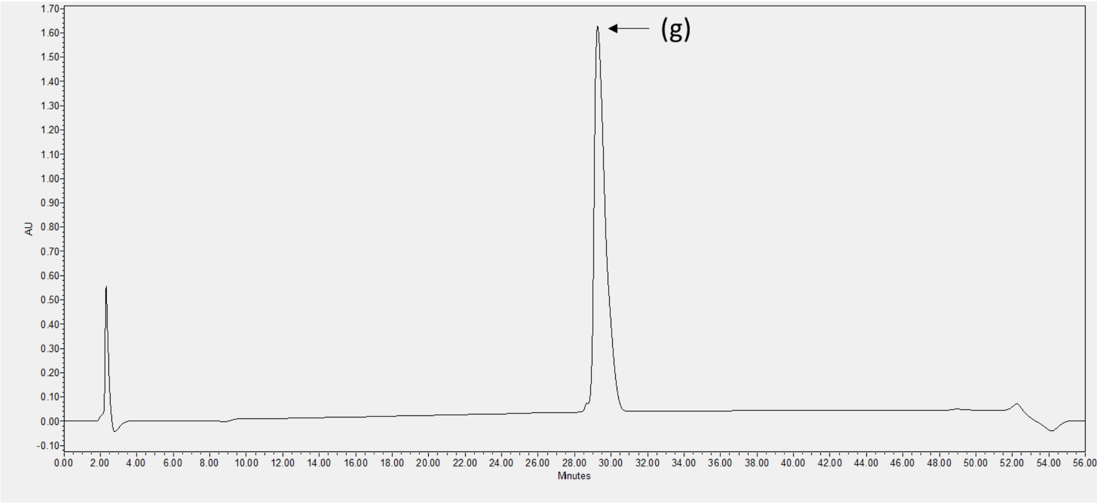

Figure S2. The mass spectra of (a) temporin-PKE, (b) temporin-PKE-2K, (c) temporin-PKE-K12, (d) temporin-PKE-3K, (e) temporin-PKE-4K, (f) temporin-PKE-i and (g) temporin-PKE-3i obtained from MALDI-TOF MS. The observed [M+H] + ions and the sodium adduct (+Na) and potassium adduct (+K) ions are indicated by arrows.

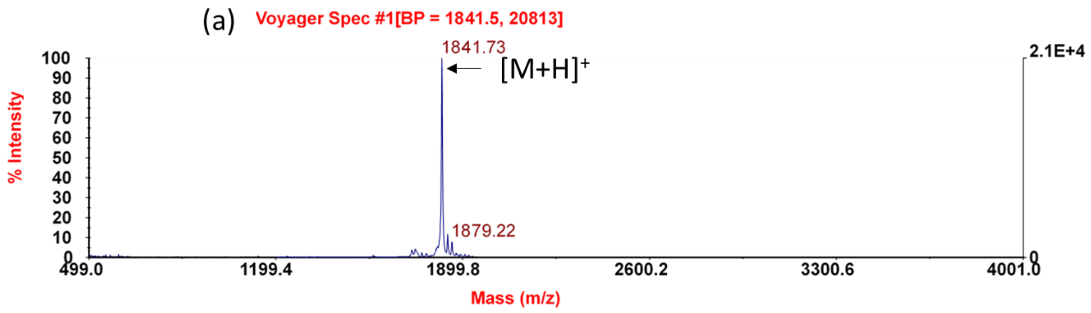

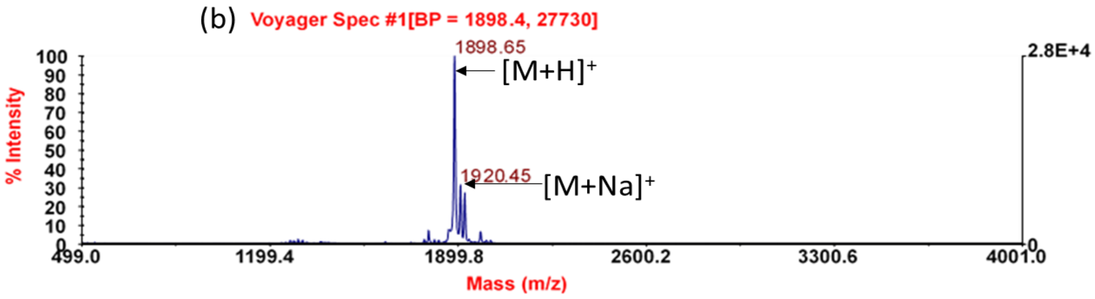

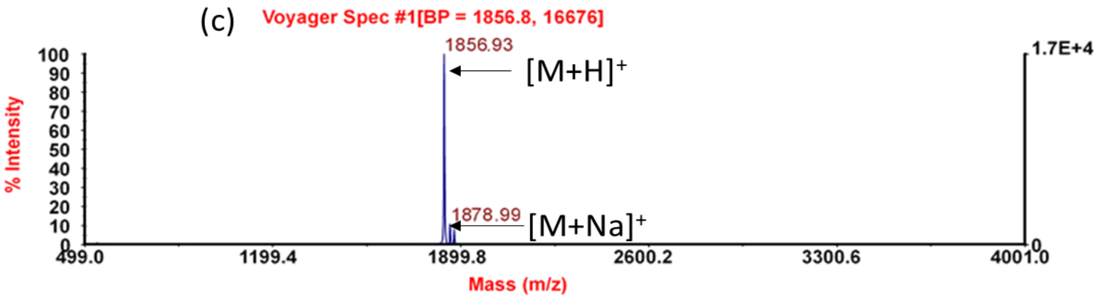

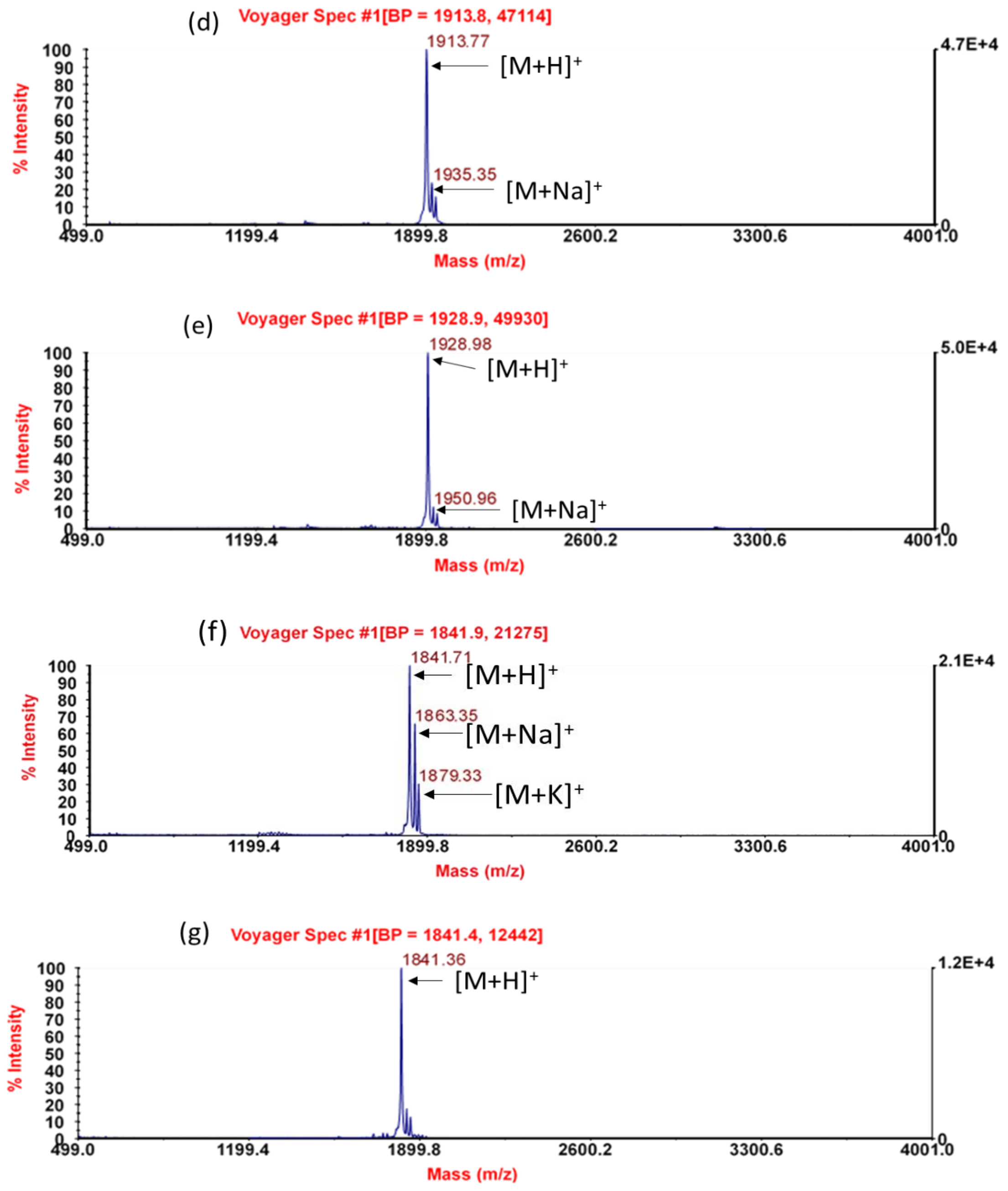
(d)
Voyager Spec #1[BP = 1913.8, 47114]
1913.77
[M+H]+
1935.35
[M+Na]+
% Intensity
4.7E+4
Mass (m/z)
(e)
Voyager Spec #1[BP = 1928.9, 49930]
1928.98
[M+H]+
1950.96
[M+Na]+
% Intensity
5.0E+4
Mass (m/z)
(f)
Voyager Spec #1[BP = 1841.9, 21275]
1841.71
[M+H]+
1863.35
[M+Na]+
1879.33
[M+K]+
% Intensity
2.1E+4
Mass (m/z)
(g)
Voyager Spec #1[BP = 1841.4, 12442]
1841.36
[M+H]+
% Intensity
1.2E+4
Mass (m/z)
499.0
1199.4
1899.8
2600.2
3300.6
4001.0
